# Impact of flipped classroom method in physical education on the intrinsic motivation, self-efficacy, and learning satisfaction: A meta-analysis

**DOI:** 10.1371/journal.pone.0324609

**Published:** 2025-05-20

**Authors:** Qingxu Wu, Zhenxiao Han, Zikang Hao, Jiping Chen, Yang Pan

**Affiliations:** 1 School of Physical Education, Shandong University, Lixia District, Jinan City, Shandong Province, China; 2 Herzen International Art College, Shandong Normal University, Lixia District, Jinan City, Shandong Province, China; University of Montenegro, MONTENEGRO

## Abstract

**Background:**

Flipped classroom (FC) is a novel teaching method. To assess its effectiveness in teaching physical education in schools, a systematic review and meta-analysis were conducted with the aim of exploring the Impact of FC method in PE on the intrinsic motivation, self-efficacy, and learning satisfaction.

**Methods:**

As of February 2025, a comprehensive search was conducted in the Web of Science (WoS) and China National Knowledge Infrastructure (CNKI) databases to identify studies pertaining to FC style PE instruction and its impact on students’ intrinsic motivation, self-efficacy, and learning satisfaction.

**Results:**

Total 13 studies were included in this study. The results demonstrated that, compared to traditional teaching methods, the FC model significantly improved intrinsic motivation, self-efficacy, and learning satisfaction. These findings underscore the notable positive impact of the FC approach on multiple educational outcomes. Subgroup analyses revealed that the FC model significantly enhanced students’ intrinsic motivation across different countries, various PE subjects, differing class sizes, and diverse student populations. In terms of learning satisfaction, the FC approach demonstrated positive effects in subgroups involving Chinese participants, PE subjects focused on dance, and varying class sizes.

**Conclusion:**

Integrating the FC model into school PE classes has been shown to enhance students’ intrinsic motivation, self-efficacy, and learning satisfaction. However, further high-quality research is essential to solidify and extend these findings.

## Introduction

In conventional educational settings, physical education (PE) instruction predominantly adheres to a teacher-centered pedagogical paradigm [1]. This approach typically imposes a uniform learning pace across the entire class, often leaving educators with insufficient time or opportunities to provide individualized guidance and support to students who encounter learning difficulties. Consequently, this one-size-fits-all approach may fail to address the diverse learning styles and preferences of students, thereby exacerbating educational disparities. Furthermore, students frequently rely on teachers’ explanations, demonstrations, and feedback, which can hinder their intrinsic motivation for autonomous exploration and active engagement in learning [2]. This dependency on external validation may limit students’ ability to develop critical thinking and problem-solving skills independently. Consequently, there is an urgent need to enhance pedagogical strategies employed in PE to better address the diverse needs of students.

In recent years, the flipped classroom (FC) model has gained traction as an innovative teaching methodology across various disciplines and has been increasingly implemented in higher education institutions [3]. Unlike traditional teaching methods, the FC adopts a student-centered approach, utilizing pre-class instructional videos to facilitate independent study, thereby freeing up valuable classroom time for more interactive and collaborative learning activities [4]. These activities may include peer interactions, teacher feedback, concept application, discussions, and problem-solving exercises [5,6]. Research indicates that this approach not only enhances students’ acquisition of knowledge and skills but also promotes personalized development and overall improvement in comprehensive abilities [3,7,8]. Although the application of FC in school PE is still in its infancy, it has gradually gained popularity in various subjects [9–11]. Preliminary evidence suggests that this approach can yield positive outcomes in terms of both learning effectiveness and student engagement [12–14]. This innovative and effective teaching approach is being increasingly adopted in the field of school PE. However, the existing research on FC in PE is primarily concerned with its impact on learning outcomes [15–17]. In comparison to the extensive research on the impact of FC-style PE on academic achievement, relatively few studies have explored the potential benefits of adopting an FC approach in PE. This is especially true concerning the psychological effects on individual students.

One of the current challenges in education is the lack of student engagement, interest, and effort in acquiring new knowledge and skills. Motivation plays a crucial role as a core driving force in the learning process. According to Self-Determination Theory (SDT) [18], motivation is a multidimensional construct that reflects varying degrees of self-determination. SDT distinguishes different types of motivation based on the reasons or goals that initiate action. SDT researchers conceptualize an individual’s motivation as a continuum ranging from intrinsic motivation to amotivation. Broadly speaking, the types of motivation reflecting behavioral engagement include intrinsic motivation, integrated regulation, and identified regulation. These forms of motivation collectively constitute autonomous motivation [19]. In contrast, motivation reflecting behaviors driven by guilt or self-imposed pressure (introjected regulation) or by external rewards or punishments (external regulation) is referred to as controlled motivation. In the school environment, the goal is for all students to achieve high levels of engagement in learning activities, leading to positive academic outcomes. Researchers have posited that self-determined motivation constitutes a higher quality of motivation, capable of yielding desirable learning outcomes [18]. Autonomous motivation is considered a crucial foundation for effective learning, with students who possess autonomous motivation often demonstrating superior performance in areas such as conceptual understanding [20].

Self-efficacy is the belief in one’s own capability to perform successfully within a field [21], while learning satisfaction is described as students’ positive evaluations of their own learning experiences [22]. Given the prevalence of significant psychological challenges among the student population [23–25], educational interventions that enhance their confidence and learning satisfaction can be highly beneficial [26,27]. It is therefore imperative to investigate the impact of FC on self-efficacy and learning satisfaction in this demographic, with a view to enhancing the overall quality of school PE.

However, the results of existing studies on the impact of FCs on intrinsic motivation [8,20,28], self-efficacy [29,30], and learning satisfaction [31,32] of student groups are inconsistent. These inconsistencies may stem from variations in study designs (e.g., sample sizes), cultural contexts, or measurement tools, leaving the true effects of FC on intrinsic motivation, self-efficacy, and satisfaction unresolved. To date, no systematic synthesis has quantitatively evaluated these mixed findings or explored potential moderators (e.g., country, sample sizes, population, subject) that may explain heterogeneity. Based on this, this study conducts a systematic review and meta-analysis of the original studies examining the effects of FC on students’ intrinsic motivation, self-efficacy and learning satisfaction, with the aim of conducting a thorough investigation of the specific impacts of FC on these outcomes.

## Methods

This systematic review was performed following the PRISMA 2020 guidelines [33], and was registered on PROSPERO (registration no. CRD42024536197).

### Data sources and search strategy

Based on prior experience [34,35], two electronic databases (Web of Science and China National Knowledge Infrastructure) were searched for controlled studies up to February 2025 on FC-style PE instruction and students’ intrinsic motivation, self-efficacy, and learning satisfaction. Eligible studies were those conducted in student populations that examined the relationship between FC-style PE instruction and at least one outcome indicator. The search was conducted independently by two researchers (Q.W. and Z.H.) who used a combination of subject terms and keywords. For example, using Web of Science as a reference, the search terms included: (flipped learning OR flipped classroom) AND (physical education OR sport* OR physical activity* OR exercise) AND (self-efficacy OR level of self-efficacy OR satisfaction OR level of satisfaction OR motivation OR learning motivation OR intrinsic motivation). Disagreements were resolved through a group discussion with a third researcher (Z.H.).

### Inclusion criteria

Detailed inclusion criteria were as follows: (1) the study design was a controlled trial; (2) the participants are student groups; (3) reported data related to the impact of a FC in PE on intrinsic motivation, self-efficacy and learning satisfaction; and (4) data were reported as means and standard deviations. In the study, uncontrolled studies, multimodal FC teaching models, and studies that failed to provide relevant data on outcomes of interest were excluded.

### Data extraction

Two researchers (Q.W. and Z.H.) independently screened the titles and abstracts using Endnote and Microsoft Excel after removing duplicates. They also independently read the full texts and hand-searched the reference lists in the relevant reviews. Disagreements were resolved by discussion with the third researcher (Z.H.). The following data were extracted from the eligible literature: first author and year of publication, country, populations, class sizes, PE subjects, research topics, required outcomes in both experimental and control groups (mean and standard deviation, SD).

### Quality appraisal

In accordance with the recommendations set forth in evidence-based medicine guidelines, the Cochrane Collaboration’s Risk of Bias Tool was employed to assess the quality of the included studies based on six criteria: (a) random sequence generation; (b) allocation concealment; (c) blinding of participants and personnel; (d)blinding of outcome assessment; (e) incomplete outcome data; (f) selective reporting; and (g) other biases [36]. For each criterion, a rating was assigned as either “high risk of bias,” “low risk of bias,” or “unclear.” This allowed for a comprehensive evaluation of the included studies in terms of their methodological quality. The process enables reviewers to evaluate the potential risk in these six areas for the purpose of ensuring the validity of the studies. Quality assessments of all studies were conducted by two independent researchers (Q.W. and J.C.). After independently rating the studies, consensus was reached through discussion, and the results were summarized in a graph.

### Statistical analysis

The methodological quality of the included studies was assessed using Review Manager 5.2 software, and meta-analyses were performed using Stata 17.0 software. Statistical significance was set at p < 0.05. The data included in this review were continuous variables, and effect sizes were expressed as standardized mean difference (SMD) [37]. Statistical heterogeneity between studies was examined using Cochrane’s Q test and the I^2^ statistic. The I^2^ statistic, with values of 25%, 50% and 75%, corresponded to low, moderate, and high level of heterogeneity, respectively [38]. To explore possible sources of heterogeneity, subgroup analyses based on country, PE subject, and class sizes were conducted. To assess the stability of the results, a sensitivity analysis was performed by recalculating the effect sizes and their 95% confidence intervals (CIs) after omitting each individual study one at a time. This approach allowed for the determination of whether any single study disproportionately influenced the overall findings. Publication bias was tested by Egger regression in Stata version 17.0, with a p-value less than 0.05 considered statistically significant for publication bias.

## Results

### Study selection

**Fig 1:**
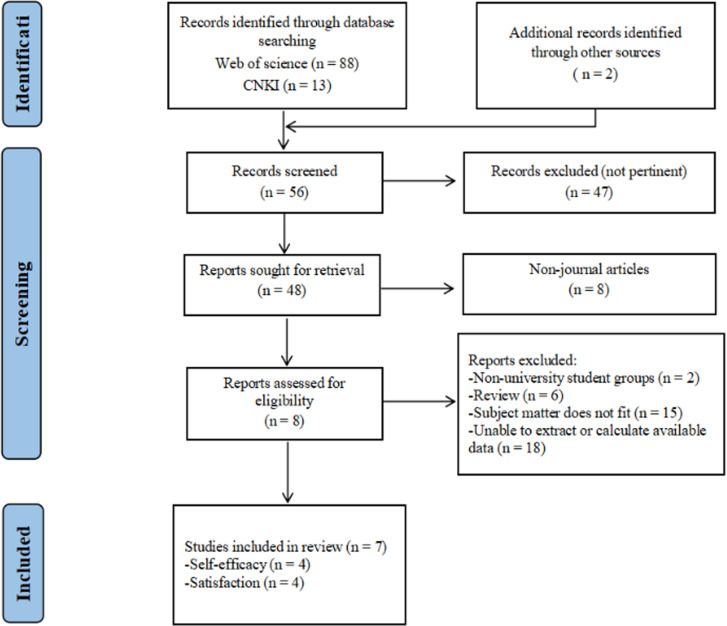
Flowchart of selection of studies included in the meta-analysis.

A total of 321 studies were initially retrieved from two databases, and 2 additional records were identified through reference checking. After a preliminary screening of titles and abstracts, 251 irrelevant studies were excluded. Subsequently, 18 non-journal articles were subsequently excluded. The remaining 54 articles were assessed by reading the full text, resulting in a final inclusion of 13 studies for the meta-analysis (Fig 1 and S1 Table).

### Quality appraisal

**Fig 2:**
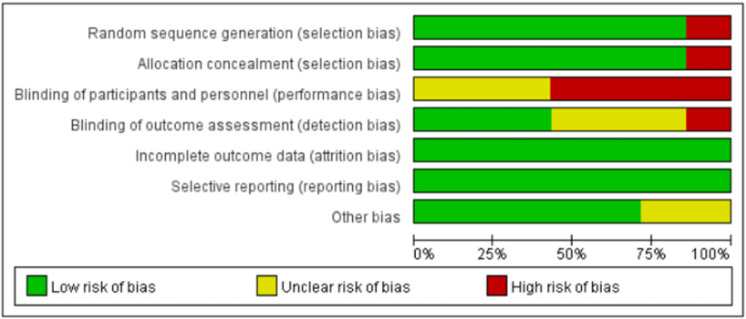
Risk bias assessment graphic for the studies included in the review.

As shown in Fig 2 and S2 Table, among the 13 studies assessed, the highest risk of bias were identified in the following areas: blinding of participants and personnel (performance bias), blinding of outcome assessment (detection bias), random sequence generation (selection bias), and allocation concealment (selection bias). An unclear risk of bias was found in several areas, including allocation concealment (selection bias), blinding of participants and personnel (performance bias), blinding of outcome assessment (detection bias), and other bias. The primary focus of the risk of bias was on blinding of participants and personnel and blinding of outcome assessment. This is mainly due to the unique nature of sports instruction methods, such as different teachers conducting the teaching, which can introduce significant variability, making it difficult to adequately blind these aspects. Overall, the quality of the original studies included was generally high.

### Characteristics of included studies

The studies were conducted in five countries: China, Spain, Norway, Indonesia, and Türkiye. Three studies focused on teaching volleyball, two on dance, two on basketball, one on endurance, strength, and coordination training, one on sprinting, one on billiards, and one on Taiji softball. Additionally, two studies did not report the subjects they taught. Eight studies examined the effect of FC instruction on students’ intrinsic motivation, four studies examined its effect on students’ self-efficacy, and four studies examined its effect on learning satisfaction (Table 1).

**Table 1:** Basic characteristics of the included studies.

Study (year)	Country	Populations	Class sizes	Subjects	Research topics	Effect size (mean±SD)	Extractor name	Date of extraction
Osterlie (2020) [20]	Norway	Secondary school	E(F): 36 C(F): 61 E(M): 49 C(M): 60	Endurance, strength and coordination	Intrinsic motivation	E(F): 5.26 ± 1.44 C(F): 4.62 ± 1.58 E(M): 5.32 ± 1.27 C(M): 4.78 ± 1.69	Q.W.	2025-01-02
Yip (2020) [28]	China	Primary school	E: 57 C: 54	Sprint	Intrinsic motivation	E: 3.27 ± 0.75 C: 3.06 ± 0.70	Z.H.	2025-01-03
Ferriz-Valero (2022) [39]	Spain	Secondary school	E: 133 C: 151	Volleyball	Intrinsic motivation	E: 4.40 ± 0.51 C: 3.77 ± 0.83	Q.W.	2025-01-04
Ridwan (2023) [40]	Indonesia	University	E(F): 8 C(F): 13 E(M): 12 C(M): 7	NR	Intrinsic motivation	E(F): 64.7 ± 4.64 C(F): 60.7 ± 3.52 E(M): 75.1 ± 11.5 C(M): 68.5 ± 7.09	Q.W.	2025-01-04
Ferriz-Valero (2022) [41]	Spain	Secondary school	E: 140 C: 123	Volleyball	Intrinsic motivation	E: 3.98 ± 0.80 C: 3.64 ± 0.80	Z.H.	2025-01-05
Lucena (2020) [42]	Spain	Primary and secondary school	E(a): 30 C(a): 30 E(b): 30 C(b): 30	NR	Intrinsic motivation	E(a): 2.73 ± 1.03 C(a): 2.00 ± 0.89 E(b): 2.66 ± 0.92 C(b): 1.87 ± 0.85	Q.W.	2025-01-06
Karaman (2023) [8]	Turkiye	Secondary school	E: 32 C: 30	Volleyball	Intrinsic motivation	E: 4.46 ± 0.55 C: 3.46 ± 0.72	Q.W.	2025-01-06
Lin (2021) [30]	China	University	E: 35 C: 40	Billiards	Self-efficacy	E: 4.25 ± 0.59 C: 4.09 ± 0.60	Z.H.	2025-01-07
					Intrinsic motivation	E: 4.22 ± 0.75 C: 3.95 ± 0.63		2025-01-07
Hu (2018) [29]	China	University	E: 71 C: 73	Basketball	Self-efficacy	E: 36.28 ± 3.814 C: 35.68 ± 3.056	Q.W.	2025-01-08
Li (2019) [43]	China	University	E: 85 C: 85	Basketball	Self-efficacy	E: 27.67 ± 3.84 C: 24.10 ± 3.52		2025-01-02
Lin (2019) [32]	China	University	E(a): 38 E(b): 38 C: 38	Dance	Self-efficacy	E(a): 3.04 ± 0.74 E(b): 3.60 ± 0.95 C: 3.04 ± 0.83	Q.W.	2025-01-08
					Learning satisfaction	E(a): 3.59 ± 0.66 E(b): 3.94 ± 0.85 C: 3.29 ± 0.65	Z.H.	2025-01-09
Chao (2021) [31]	China	University	E(a): 68 C(a): 58 E(b): 21 C(b): 19	Dance	Learning satisfaction	E(a): 18.54 ± 2.54 C(a): 17.28 ± 3.27 E(b): 18.52 ± 3.06 C(b): 17.63 ± 3.40	Z.H.	2025-01-09
Li (2018) [44]	China	University	E: 60 C: 58	Taiji softball	Learning satisfaction	E: 91.14 ± 7.28 C: 82.46 ± 6.28	Z.H.	2025-01-09

a or b: different subgroups in the same study; C: control group; E: experimental group; F: female; SD, standard deviation; M: male; NR, not reported.

### Intrinsic motivation

**Fig 3:**
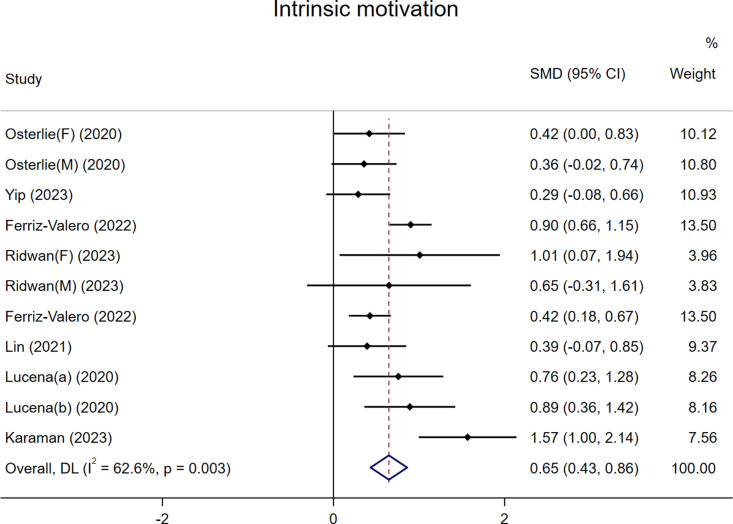
The effect of FC versus traditional PE on students’ intrinsic motivation.

As shown in Fig 3, a total of eight studies comprising 11 datasets were included in the meta-analysis examining the impact of FC on students’ intrinsic motivation [8,20,28,30,39–42]. The results indicated that FC in PE significantly enhanced students’ intrinsic motivation, with a SMD of 0.65 (p < 0.05). However, the results exhibited moderate heterogeneity (I^2^ = 62.6%, p = 0.003). To explore potential sources of this heterogeneity and verify the robustness of the findings, subgroup analyses were conducted based on country, student population, teaching subjects, and the class sizes (S1–4 Figs). The results revealed that FC significantly improved students’ intrinsic motivation across different countries, PE subjects, class sizes, and student populations. None of these factors were identified as significant contributors to the observed heterogeneity. Subsequently, a leave-one-out sensitivity analysis was performed to assess the stability of the findings. The results showed that after excluding any single study, neither the overall effect size nor its 95% CI changed substantially, indicating the robustness of our findings (S5 Fig).

### Self-efficacy

**Fig 4:**
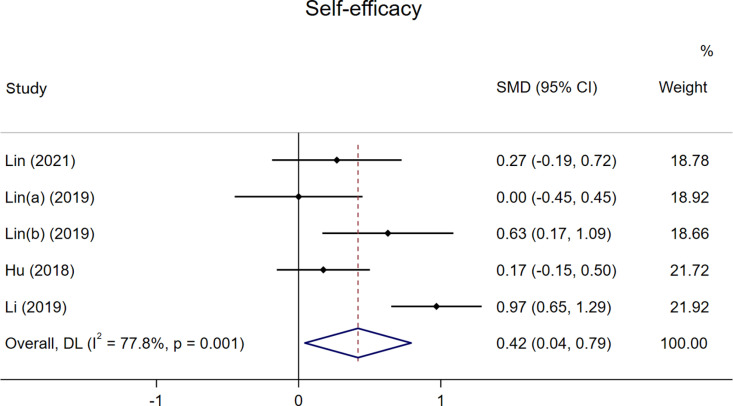
The effect of FC versus traditional PE on students’ self-efficacy.

As shown in Fig 4, a total of four studies were included in this analysis [29,30,32,43], with the study by Lin et al. [32] providing two sets of data. The results indicated a significant positive effect of FC on students’ self-efficacy compared to traditional PE teaching, with a SMD of 0.42 (p < 0.05). However, the results exhibited high heterogeneity (I^2^ = 77.8%, p = 0.001).

Given that all included studies were conducted in China and involved college student populations, subgroup analyses were performed based on teaching subjects and the class sizes (S6–7 Figs). These factors were not identified as significant contributors to the observed high heterogeneity. A subsequent leave-one-out sensitivity analysis demonstrated that after excluding any single study, neither the overall effect size nor its 95% CI changed substantially (S8 Fig). Furthermore, the studies included in this meta-analysis were of high quality, which enhances the reliability of our findings.

### Learning satisfaction

**Fig 5:**
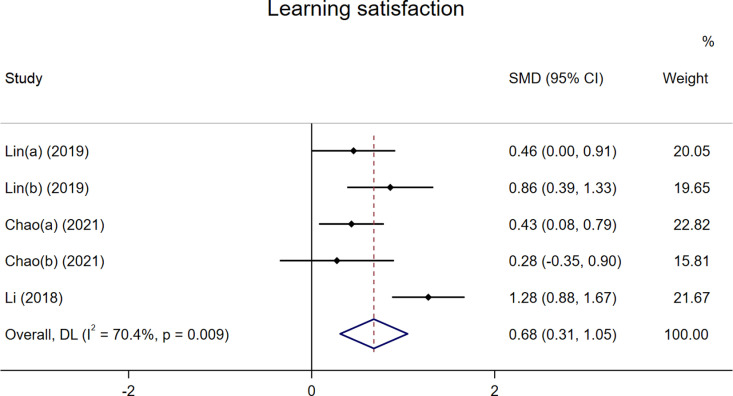
The effect of FC versus traditional PE on students’ learning satisfaction.

A total of three studies were included in the analysis of learning satisfaction [31,32,44], with two datasets provided by Lin et al. [32]. As shown in Fig 5, the results indicated a significant positive effect of FC on students’ learning satisfaction compared to traditional PE teaching, with a SMD of 0.68 (p < 0.05). However, there was high heterogeneity (I^2^ = 70.4%, p = 0.009).

Subgroup analyses were conducted based on country, PE subjects, and number of participants (S9–11 Figs). The results indicated that in studies involving participants exclusively from China, the FC approach significantly enhanced students’ learning satisfaction. Notably, after excluding ball sports from the analysis, the heterogeneity disappeared, and the results remained significant. Therefore, PE subjects appear to be a primary source of the observed heterogeneity.

The analysis related to the class sizes showed that FC significantly improved students’ learning satisfaction in both smaller classes (≤40) and larger classes (>40). Additionally, a leave-one-out sensitivity analysis demonstrated that the findings were stable after excluding any single study (S12 Fig), indicating that the positive impact of FC on students’ academic satisfaction is robust

### Publication bias

The meta-analysis of intrinsic motivation, self-efficacy, and learning satisfaction employed funnel plots and Egger’s test to assess publication bias. Visual inspection of funnel plots for asymmetry was conducted (S13–15 Figs). Additionally, Egger’s test was performed to statistically evaluate the presence of publication bias (S3 Table). The results indicated no evidence of publication bias.

## Discussion

This review study has identified the positive effects of FC instruction in PE on students’ intrinsic motivation, self-efficacy, and learning satisfaction. Compared to traditional sports teaching methods, FC has demonstrated a significant improvement in students’ intrinsic motivation (SMD = 0.65), self-efficacy (SMD = 0.42), and learning satisfaction (SMD = 0.68). Additionally, subgroup analyses revealed that FC significantly increased students’ intrinsic motivation across different countries, PE subjects, student populations, and class sizes. Regarding learning satisfaction, FC significantly improved students’ learning satisfaction regardless of the number of participants.

In recent years, the FC has emerged as an innovative pedagogical approach in PE, demonstrating significant potential. Empirical studies have shown that FC positively impacts students’ motor skill acquisition, motivation, and social competence. For instance, in primary school PE settings, students exposed to FC outperformed their counterparts in traditional teaching models. The high engagement characteristic of FC not only facilitates the development of new motor skills but also enhances students’ social competencies, transforming them into active agents of their learning process [45]. Furthermore, FC implementation has been shown to significantly improve elementary students’ understanding of rules and game strategies [46]. Studies by Botella [47]and Ferriz-Valero [39], among others, further confirmed that FC significantly enhances students’ intrinsic and autonomous motivation. This leads to more active participation in classroom activities and a perception that the learning process is more interesting and productive. Additionally, Zhao et al. [48] demonstrated the effectiveness of FC in table tennis club courses, highlighting its capacity to improve students’ problem identification, analysis, and resolution skills, thereby embodying the student-centered teaching philosophy.

Despite these valuable insights, several limitations persist in current research. First, substantial heterogeneity in sample sizes and methodologies across studies raises concerns about the consistency and reliability of findings. Second, existing research predominantly focuses on specific PE subjects, lacking a comprehensive perspective. Therefore, this study aimed to address these gaps by focusing on the effects of FC on students’ intrinsic motivation, self-efficacy, and learning satisfaction through a systematic review and meta-analysis.

First, our study found that FC significantly enhanced students’ intrinsic motivation. This finding supports previous research indicating an increase in students’ intrinsic and/or extrinsic motivation when FC is implemented in educational settings [5,49]. Consistent with this, it is widely acknowledged that students who derive enjoyment and satisfaction from their activities tend to exhibit greater enthusiasm and engagement in those tasks [50], which in turn improves their learning strategies and academic performance [51,52]. However, conflicting results have also been reported in the literature. For instance, Campos-Gutiérrez et al. [53] did not find a significant positive effect of FC on intrinsic motivation. This discrepancy may be attributed to the relatively short duration of the FC intervention or the brief time interval between the intervention and the administration of the questionnaire. Similarly, Osterlie et al. [20] revealed differences in the impact of FC on intrinsic motivation across genders. Specifically, boys appeared to benefit more from FC than girls. A plausible explanation for this finding is that the activities introduced through FC may align more closely with contexts traditionally dominated by boys, thereby increasing its perceived relevance for male students. However, this does not imply that FC is less important for girls. Therefore, further investigation is warranted to explore whether FC has differential effects on intrinsic motivation across genders. Additionally, the subgroup analyses revealed that FC significantly improved intrinsic motivation across different countries, class sizes, and student populations. Notably, in smaller classes (≤40), the effect size was larger (SMD = 0.87). This enhanced effect in smaller classes may be attributed to teachers’ ability to better tailor instruction to individual student differences, providing more personalized guidance [54–56]. Additionally, superior classroom management, closer peer relationships, and more flexible instructional arrangements likely contribute to increased intrinsic motivation among students. Future research should aim to supplement this field with high-quality evidence. Moreover, some studies have found that gamified functional lessons may have a more significant positive impact on learning motivation, which seems to be one of the main directions for future research [57,58]. In summary, the results of this study on FC and student motivation, combined with the predominantly positive outcomes observed, indicate that the implementation of the FC approach effectively increases students’ motivation.

In terms of self-efficacy, this study also identified a significant positive impact of the FC model. Sensitivity analysis further confirmed the robustness and reliability of this finding. The positive effect of FC on self-efficacy has also been observed in other educational domains [59–61]。In PE, FC may enhance students’ self-efficacy by assigning specific roles and responsibilities tailored to individual students [62]. For example, assigning appropriate positions to students during team sports activities can contribute to this effect. Students have reported placing great importance on their assigned roles within the team, as these roles provide them with greater autonomy during the collaborative learning process [63]. This aligns with previous research suggesting that providing students with more opportunities to apply theoretical knowledge in practical classroom settings can significantly enhance their self-efficacy [64]. Furthermore, because PE is a relatively complex discipline encompassing various subjects, some of which have minimal overlap, the inherent characteristics of different subjects may lead to variations in effect size. For example, in basketball, a successful shot can immediately lead to greater self-satisfaction. In addition, even if the students are at a lower level, a true leader on the team can create a positive atmosphere, which increases the mental capital and engagement of the rest of the team, which is critical to developing students’ self-efficacy [65,66]. Conversely, for dance students, psychological and cognitive factors, as well as issues related to injuries, academic stress, and negative body image, may negatively affect their self-efficacy [67,68].

Meta-analyses from non-PE disciplines have demonstrated that the FC model can enhance students’ motivation, self-efficacy and learning satisfaction [59,69–72], which is consistent with the findings of this study. However, there are also studies in other educational domains reporting findings inconsistent with our results. For instance, van Alten et al. [12] found that the impact of FC on student satisfaction was not statistically significant, with an effect size close to zero. Similar results were found in the study by Shi et al. [73]. Similarly, a meta-analysis on blended learning reported a negligible effect size (g = 0.11) on student satisfaction, which also failed to reach statistical significance [74]. In contrast, within PE, current research tends to support the positive impact of FC on improving students’ learning satisfaction [10,50]. The discrepancies in these findings may be attributed to students’ perceptions that online learning requires a greater time commitment compared to traditional classroom instruction [75]. Additionally, increased workload has been identified as one of the most frequently cited concerns among students in flipped classrooms [76]. However, in PE, particularly in flipped PE settings, the extent of increased workload may not be as pronounced as in other subjects. This is because PE inherently involves a substantial amount of physical activity and hands-on practice, which already demands significant physical and temporal engagement. Consequently, compared to other disciplines, students in PE may not need to invest excessive additional time in online learning or completing supplementary assignments when participating in flipped classrooms. Interestingly, subgroup analysis revealed that the positive impact of FC on learning satisfaction becomes more pronounced as class size increases. This finding aligns with the discussion above, especially in the context of PE, where larger class sizes may facilitate task distribution among more students, thereby significantly reducing the individual workload per student. Furthermore, enhanced peer interaction driven by diverse skill levels and learning styles may also contribute to this effect. Increased opportunities for peer teaching, collaboration, and comprehensive feedback networks among students could be additional factors supporting this outcome [77,78]. However, it is important to note that these potential benefits are contingent upon the proper implementation of the FC methodology, including adequate technological infrastructure, appropriate instructor training, and effective classroom management strategies.

This study has several limitations. (1) The majority of the included studies were conducted in China, and there is a lack of research on student self-efficacy and learning satisfaction from other countries. (2) Several of the results displayed notable heterogeneity, and we were unable to identify the precise sources of this variability. Nevertheless, the overall quality of the included studies was high, and the leave-one-out sensitivity analysis revealed that excluding any single study did not lead to significant changes in the overall effect size or its 95% CI. These findings highlight the robustness and reliability of our results. (3) Flipped PE instruction is still in its exploratory phase, which resulted in a limited number of studies included in this review and there is still a lack of more recent and relevant research. Future research should aim to include more PE subjects and higher-quality studies to further expand and enrich the evidence base.

## Conclusion

The integration of information and communication technologies into educational practices has become increasingly prevalent, necessitating the innovation and adaptation of pedagogical approaches across all school subjects, including PE. The FC model has emerged as a promising instructional strategy in PE, offering potential benefits for student learning and motivation. This study provides evidence that the FC approach significantly enhances students’ intrinsic motivation, self-efficacy, and learning satisfaction compared to traditional PE teaching methods. One of the key strengths of this study lies in its comprehensive meta-analytic approach, which synthesizes data from multiple studies across different countries and PE subjects, providing a robust evaluation of the FC model’s effectiveness. However, certain limitations should be acknowledged. The heterogeneity observed in some of the results, particularly in self-efficacy and learning satisfaction, suggests that further research is needed to identify the specific factors contributing to these variations. However, the sensitivity analyses indicate that the results of this study are reliable. We encourage further research to expand the scope of this study by including more diverse populations, exploring additional PE subjects, and examining the long-term effects of the FC model on student achievement.

## Supporting information

S1 Appendix Additional results and data. (DOCX)
