# Supplementary material for: Impact of flipped classroom method in physical education on the intrinsic motivation, self-efficacy, and learning satisfaction: A meta-analysis
Source: PLoS One. 2025 May 20;20(5):e0324609. doi: 10.1371/journal.pone.0324609 (PMC12091796; doi:10.1371/journal.pone.0324609)
Supplement: S1 Appendix — (DOCX) [file pone.0324609.s001.docx]

| **S1 Table. A numbered table of studies identified in the literature search.** | | | | |
| --- | --- | --- | --- | --- |
| **Study ID** | **Author (year)** | **Title** | **Included/Excluded** | **Reasons for exclusion** |
| 1 | Osterlie (2020) | The Impact of Flipped Learning on Cognitive Knowledge Learning and Intrinsic Motivation in Norwegian Secondary Physical Education | Included |  |
| 2 | Yip (2020) | The association of children's motivation and physical activity levels with flipped learning during physical education lessons | Included |  |
| 3 | Ferriz-Valero (2022) | Flipped Classroom: A Good Way for Lower Secondary Physical Education Students to Learn Volleyball | Included |  |
| 4 | Ridwan (2023) | Flipped Learning as a Solution to Increase Motivation and Academic Achievement: Five Weeks of Mixed Research | Included |  |
| 5 | Ferriz-Valero (2022) | The Effects of Flipped Learning on Learning and Motivation of Upper Secondary School Physical Education Students | Included |  |
| 6 | Lucena (2020) | Academic Effects of the Use of Flipped Learning in Physical Education | Included |  |
| 7 | Karaman (2023) | The Effect of Flipped Learning Physical Education on Students' Knowledge, Skills and Motivation | Included |  |
| 8 | Lin (2021) | Promoting pre-class guidance and in-class reflection: A SQIRC-based mobile flipped learning approach to promoting students' billiards skills, strategies, motivation and self-efficacy | Included |  |
| 9 | Hu (2018) | An experimental study on the application of flipped classroom teaching mode to public physical education courses in general colleges and universities | Included |  |
| 10 | Li (2019) | A Practical Study of Flipped Classroom Based on WeChat Public Platform in Basketball Option Class in Our Schools | Included |  |
| 11 | Lin (2019) | Effects of integrating mobile technology-assisted peer assessment into flipped learning on students' dance skills and self-efficacy | Included |  |
| 12 | Chao (2021) | Do socio-cultural differences matter? A study of the learning effects and satisfaction with physical activity from digital learning assimilated into a university dance course | Included |  |
| 13 | Li (2018) | Application Study on the Flipped Classroom in the Taiji Softball Teaching in Colleges and Universities | Included |  |
| 14 | Hu (2022) | The influence of "small private online course plus flipped classroom" teaching on physical education students' learning motivation from the perspective of self-determination theory | Excluded | Subject matter does not fit |
| 15 | Segura-Robles (2020) | Effects on Personal Factors Through Flipped Learning and Gamification as Combined Methodologies in Secondary Education | Excluded | Unable to extract or calculate available data |
| 16 | AE (2014) | A case study of Flipped Learning at College: Focused on Effects of Motivation and Self-efficacy | Excluded | Subject matter does not fit |
| 17 | Qu (2022) | Improving Students' Learning Motivation and Attitude in Physical Education by Using Algorithm Thinking Innovation Teaching Method | Excluded | Records excluded (not pertinent) |
| 18 | Ünal (2024) | Unlocking Potential: Assessing Motivation, Learning Strategies, and Cognitive Load in Flipped Learning during Online Teaching in Higher Education | Excluded | Unable to extract or calculate available data |
| 19 | Vaughn (2014) | The Effectiveness of a Flipped Classroom Approach Unit on Student Knowledge, Skill Development, and Perception of Collegiate Physical Education | Excluded | Records excluded (not pertinent) |
| 20 | Covill (2019) | Comparison of Academic Performance in Traditional and Flipped Classrooms and Students' Attitudes of the Flipped Experience | Excluded | Unable to extract or calculate available data |
| 21 | Sailer (2021) | Gamification of in-class activities in flipped classroom lectures | Excluded | Records excluded (not pertinent) |
| 22 | Botella (2021) | Flipped Learning to improve students' motivation in Physical Education | Excluded | Unable to extract or calculate available data |
| 23 | Kim (2023) | The Effect of Flipped Learning Education on Academic Motivation and Class Satisfaction in Physical Therapy Students | Excluded | Records excluded (not pertinent) |
| 24 | Lin (2019) | An online peer assessment approach to supporting mind-mapping flipped learning activities for college English writing courses | Excluded | Records excluded (not pertinent) |
| 25 | Frew (2023) | An Examination of Physical Literacy: Learning Through a Technology Integrated, Flipped Classroom Approach | Excluded | Records excluded (not pertinent) |
| 26 | sun-mi (2021) | Application of Flipped Learning in a Liberal French Conversation Course | Excluded | Records excluded (not pertinent) |
| 27 | Akers (2021) | Flipping the PE Classroom to Increase Physical Activity | Excluded | Records excluded (not pertinent) |
| 28 | Vidal (2024) | Level of Satisfaction with the Application of the Collaborative Model of the Flipped Classroom in the Sport of Sailing | Excluded | Unable to extract or calculate available data |
| 29 | Breitkreuz (2020) | Impact of Flipped Classroom Model on Student Learning Outcomes for University Fitness/Wellness Learners | Excluded | Records excluded (not pertinent) |
| 30 | Osterlie (2019) | The Perception of Adolescents' Encounter With a Flipped Learning Intervention in Norwegian Physical Education | Excluded | Records excluded (not pertinent) |
| 31 | Campos-Gutiérrez (2021) | FLIPPED LEARNING IN PHYSICAL EDUCATION: LEARNING, MOTIVATION AND MOTOR PRACTICE TIME | Excluded | Unable to extract or calculate available data |
| 32 | Tien (2019) | Applying Structured Computer-Assisted Collaborative Concept Mapping to Flipped Classroom | Excluded | Non-journal article |
| 33 | Díaz-Alvarez (2023) | "FLIPPED ASSESSMENT": Proposal for a Self-Assessment Method to Improve Learning in the Field of Manufacturing Technologies | Excluded | Records excluded (not pertinent) |
| 34 | Galvez (2019) | IMPLEMENTATION AND EVALUATION OF FLIPPED LEARNING FOR DELIVERY OF PLANT PROTECTION TOPICS | Excluded | Records excluded (not pertinent) |
| 35 | Ridwan (2023) | Flipped Learning as a Solution to Increase Motivation and Academic Achievement: Five Weeks of Mixed Research | Excluded | Records excluded (not pertinent) |
| 36 | Gosálbez-Carpena (2022) | METHODOLOGICAL APPLICATION FLIPPED CLASSROOM IN THE NON-UNIVERSITY STUDENTS OF PHYSICAL EDUCATION: A SYSTEMATIC REVIE | Excluded | Review |
| 37 | Panissa (2021) | Teaching Exercise Physiology with Flipped Classroom Method in The Era of COVID-19: Experience of a Remote Course | Excluded | Subject matter does not fit |
| 38 | Kim (2017) | ANALYSIS OF LEARNING EFFECT OF FLIPPED LEARNING BASED COMPUTER USE LESSON | Excluded | Records excluded (not pertinent) |
| 39 | Ramazanoglu (2025) | The Effect of Peer Mentoring on Web 2.0 Usage Competence and Self-Efficacy Beliefs in Flipped Classrooms | Excluded | Records excluded (not pertinent) |
| 40 | Ruano (2021) | Comparative analysis of motivational profiles and flow status between a traditional methodology and the Flipped Classroom methodology in Physical Education students | Excluded | Unable to extract or calculate available data |
| 41 | Matsumoto (2016) | FLIPPED CLASSROOM USING GAMIFICATION | Excluded | Records excluded (not pertinent) |
| 42 | Di (2016) | The Application of Flipped Classroom Model in Dragon Dance Training in University-8 Tracing Circle Dance Dragon Teaching as an Example | Excluded | Subject matter does not fit |
| 43 | Chiou (2020) | Applying structured computer-assisted collaborative concept mapping to flipped classroom for hospitality accounting | Excluded | Records excluded (not pertinent) |
| 44 | Malmquist (2016) | Novel Graduated Engagement Strategy Tools for Enhancing Student Success in a Large Diverse Introductory Physiology Course | Excluded | Records excluded (not pertinent) |
| 45 | Mazana (2020) | Flipping college mathematics classroom | Excluded | Records excluded (not pertinent) |
| 46 | Koh (2021) | Preservice Physical Education Teachers' Perceptions of a Flipped Basketball Course: Benefits, Challenges, and Recommendations | Excluded | Subject matter does not fit |
| 47 | Khoiriyah (2017) | Promoting Flipped Classroom Model in Teaching Writing of EFL Learners | Excluded | Records excluded (not pertinent) |
| 48 | Cuervo (2021) | Implementation of the Flipped Classroom methodology in the subject "Legal and Professional Context of Physical Activity and Sport". Implications on the perception, motivation and student academic results | Excluded | Subject matter does not fit |
| 49 | Vasilchenko (2020) | Engaging Science and Engineering Students in Computing Education through Learner-Created Videos and Physical Computing Tools | Excluded | Records excluded (not pertinent) |
| 50 | Reinoso (2021) | Effect of flipped teaching on the performance and perceptions of pre-service teachers on a biology course | Excluded | Records excluded (not pertinent) |
| 51 | Kaya (2025) | Unleashing the potential of flipped learning in K-12: A review of experimental studies | Excluded | Review |
| 52 | Yiu (2019) | Flip Fashion Studies Classroom: Students’ Perception and Motivation | Excluded | Records excluded (not pertinent) |
| 53 | Chiang (2017) | Investigating the motivation between ubiquitous learning strategy and gender for basketball sport literacy | Excluded | Subject matter does not fit |
| 54 | Hinojo-Lucena (2018) | Incidence of the Flipped Classroom in the Physical Education Students' Academic Performance in University Contexts | Excluded | Subject matter does not fit |
| 55 | Yang (2021) | Research focuses and findings of flipping mathematics classes: a review of journal publications based on the technology-enhanced learning model | Excluded | Records excluded (not pertinent) |
| 56 | Chao (2021) | Exploring the Effects of Blended Learning, Flipped Learning, and Online Remedial Teaching on Improving Students' Learning Performance and Motivation | Excluded | Records excluded (not pertinent) |
| 57 | Kwong (2024) | Autonomous and controlled motivation in a flipped-classroom approach | Excluded | Records excluded (not pertinent) |
| 58 | Ghorbel (2025) | Flipped classroom approach for gymnastics learning in physical education: A quasi-experimental study | Excluded | Unable to extract or calculate available data |
| 59 | Malmquist (2017) | Interactions Between Student Attitudes, Affect and Outcomes During a Transition to an Active Learning Format in Introductory Physiology | Excluded | Subject matter does not fit |
| 60 | Araujo (2024) | Education of 21st century: A proposal of flipped classroom strategy to teach Soil Biology | Excluded | Records excluded (not pertinent) |
| 61 | Peña-González (2023) | The effect of a combination of flipped classroom and gamification on university student's perceived teaching quality, subject satisfaction and academic performance | Excluded | Unable to extract or calculate available data |
| 62 | Cho (2016) | Effect of Flipped Learning Education in Physical Examination and Practicum | Excluded | Records excluded (not pertinent) |
| 63 | Vasilchenko (2020) | Engaging Science and Engineering Students in Computing Education through Learner-Created Videos and Physical Computing Tools | Excluded | Records excluded (not pertinent) |
| 64 | Wen-Chun (2017) | The research of applying mobile virtual reality to martial arts learning system with flipped classroom | Excluded | Unable to extract or calculate available data |
| 65 | Sevillano-Monje (2022) | The Flipped Classroom and the Development of Competences: A Teaching Innovation Experience in Higher Education | Excluded | Records excluded (not pertinent) |
| 66 | Gómez-García (2020) | Effect of the Flipped Classroom and Gamification Methods in the Development of a Didactic Unit on Healthy Habits and Diet in Primary Education | Excluded | Subject matter does not fit |
| 67 | Mohammed (2021) | Analytic Hierarchy Process for Evaluating Flipped Classroom Learning | Excluded | Records excluded (not pertinent) |
| 68 | Etemi (2020) | The Effects of Flipped Learning Method on Students' Perception and Learning of Java Programming | Excluded | Records excluded (not pertinent) |
| 69 | Mazana (2020) | Flipping college mathematics classroom | Excluded | Records excluded (not pertinent) |
| 70 | Ma (2024) | A full-flipped classroom mode from the perspective of Junior High School English teachers | Excluded | Records excluded (not pertinent) |
| 71 | Ng (2016) | Are my Students Ready: A Case of Flipped Learning in an IT Subject | Excluded | Records excluded (not pertinent) |
| 72 | Ghafouri (2024) | Comparison of education using the flipped class, gamification and gamification in the flipped learning environment on the performance of nursing students in a client health assessment: a randomized clinical trial | Excluded | Records excluded (not pertinent) |
| 73 | Dolezal (2018) | Effects of Introducing a Game-Based Student Response System into a Flipped, Person-Centered Classroom on Object-Oriented Design | Excluded | Records excluded (not pertinent) |
| 74 | Elliott (2014) | Do Students Like the Flipped Classroom? An Investigation of Student Reaction to a Flipped Undergraduate IT Course | Excluded | Records excluded (not pertinent) |
| 75 | Eichler (2022) | Future of the Flipped Classroom in Chemistry Education: Recognizing the Value of Independent Preclass Learning and Promoting Deeper Understanding of Chemical Ways of Thinking During In-Person Instruction | Excluded | Records excluded (not pertinent) |
| 76 | Suwapaet (2018) | Introducing a Flipped Classroom to Engineering Students: A Case Study in Mechanics of Materials Course | Excluded | Records excluded (not pertinent) |
| 77 | Roe (2019) | Learning with technology in physiotherapy education: design, implementation and evaluation of a flipped classroom teaching approach | Excluded | Records excluded (not pertinent) |
| 78 | Ferzli (2015) | DEVELOPMENT OF A GAMIFIED LEARNING OBJECT (GLO) WITH CORRELATED CLASSROOM ACTIVITIES TO ENHANCE STUDENT UNDERSTANDING OF EVOLUTION | Excluded | Records excluded (not pertinent) |
| 79 | Carey (2023) | Here's an IDEA to Improve Sport Education: Use a Flipped Classroom to Increase Student Role Efficacy | Excluded | Unable to extract or calculate available data |
| 80 | Mennella (2016) | Comparing the Efficacy of Flipped vs. Alternative Active Learning in a College Genetics Course | Excluded | Records excluded (not pertinent) |
| 81 | Pitt (2022) | Dependence of learning outcomes in flipped and lecture classrooms on review questions: A randomized controlled trial and observational study | Excluded | Records excluded (not pertinent) |
| 82 | Ahmed (2024) | The Efficacy of Flipped Learning in Boosting Children's Motivation and Physical Activity During Physical Education Sessions | Excluded | Subject matter does not fit |
| 83 | Osterlie (2023) | Flipped learning in physical education: A scoping review | Excluded | Review |
| 84 | Xiaofang (2020) | The construction and application of MOOC + flipped classroom teaching model for table tennis in colleges and universities | Excluded | Subject matter does not fit |
| 85 | Gastardo (2018) | EXPLORING THE FUNCTIONALITY OF LEARNING ANALYTICS IN FLIPPED LESSONS WITH INTEGRATED VIDEO IN AN UNDERGRADUATE MATHEMATICS COURSE | Excluded | Records excluded (not pertinent) |
| 86 | Custodio (2021) | Empirical didactic experience about flipped classroom on Physical Education area | Excluded | Subject matter does not fit |
| 87 | Meana (2021) | Flipped Classroom and gamification in Automated Manufacturing lab classes | Excluded | Records excluded (not pertinent) |
| 88 | Harris (2024) | Student comprehension of biochemistry in a flipped classroom format | Excluded | Records excluded (not pertinent) |
| 89 | Casas-Moreno (2022) | THE FLIPPED CLASSROOM FOR TEACHING JUDO IN PHYSICAL EDUCATION | Excluded | Subject matter does not fit |
| 90 | Vanaclocha-Amat (2025) | RETRAGAM: Resistance training based on gamification during physical education. Rationale and study protocol | Excluded | Subject matter does not fit |
| 91 | Soriano-Pascual (2022) | Flipped Classroom through Edpuzzle® and the sports teaching-learning process in secondary school students: A pilot study | Excluded | Subject matter does not fit |
| 92 | Thulasidas (2024) | Flipped Classroom for Linear Algebra at Undergraduate Level | Excluded | Records excluded (not pertinent) |
| 93 | Rodríguez (2016) | FLIPPED LEARNING MODEL AT TRAINING SECONDARY TEACHERS. TRAINING FOR CHANGE | Excluded | Records excluded (not pertinent) |
| 94 | Thulasidas (2024) | Flipped Classroom for Linear Algebra at Undergraduate Level | Excluded | Records excluded (not pertinent) |
| 95 | Schäfer (2019) | Teaching Modern C plus plus with Flipped Classroom and Enjoyable IoT Hardware | Excluded | Records excluded (not pertinent) |
| 96 | Wehling (2024) | Providing practical skills in curricular teaching-effect of SkillsLab and flipped classroom | Excluded | Records excluded (not pertinent) |
| 97 | Umetani (2023) | Artificial-Voice-Based Conversational Lecture Video Clips for Flipped Classroom | Excluded | Records excluded (not pertinent) |
| 98 | Watson (2022) | Design, implementation, and insights from a volcanology Virtual Field Trip to Iceland | Excluded | Records excluded (not pertinent) |
| 99 | Qutob (2022) | Effect of flipped classroom approach in the teaching of a hematology course | Excluded | Records excluded (not pertinent) |
| 100 | Graff (2023) | Occupational Therapy Student Perceptions of Team-Based Learning and Flipped Classroom Learning Activities | Excluded | Records excluded (not pertinent) |
| 101 | Armengol (2023) | Feed Back as a Teaching Tool: Its Impact on the Motivation of Higher Education Students | Excluded | Review |
| 102 | Prevalla (2019) | Flipped Learning in Engineering Education | Excluded | Records excluded (not pertinent) |
| 103 | Balan (2015) | Preparing students for Flipped or Team-Based Learning methods | Excluded | Records excluded (not pertinent) |
| 104 | Schafer (2019) | Teaching Modern C++ with Flipped Classroom and Enjoyable IoT Hardware | Excluded | Records excluded (not pertinent) |
| 105 | Gedik (2024) | Investigation of Student Experinces in Flipped Programming Using Epistemic Network Analysis | Excluded | Records excluded (not pertinent) |
| 106 | Elliott (2014) | Impact of increased photovoltaic generation on inter-area oscillations in the Western North American power system | Excluded | Records excluded (not pertinent) |
| 107 | Ossovski (2022) | Digitally Supported Introductory University Teaching in Computer Science Considering Heterogeneous Groups | Excluded | Records excluded (not pertinent) |
| 108 | Zhong (2022) | Effectiveness comparison between blended learning of histology practical in flipped physical classrooms and flipped virtual classrooms for MBBS students | Excluded | Records excluded (not pertinent) |
| 109 | Zupanec (2018) | DETERMINATION OF EDUCATIONAL EFFICIENCY AND STUDENTS' INVOLVEMENT IN THE FLIPPED BIOLOGY CLASSROOM IN PRIMARY SCHOOL | Excluded | Records excluded (not pertinent) |
| 110 | Qin (2022) | Unpacking the interaction between foreign language learners' emotion, cognition, and activity in the flipped classroom in higher education: A perezhivanie perspective | Excluded | Records excluded (not pertinent) |
| 111 | Gross (2018) | Implementation of an Inverted Classroom in Structural Design Courses | Excluded | Records excluded (not pertinent) |
| 112 | Min-Chae (2022) | Diagnosis of Competency of Preliminary Special Physical Education Teachers according to Participation in Flipped Learning Application Class | Excluded | Subject matter does not fit |
| 113 | Capella-Peris (2023) | How to Promote Social Entrepreneurship on Urban Education? An Active Learning Implementation | Excluded | Records excluded (not pertinent) |
| 114 | Sajid (2016) | Can blended learning and the flipped classroom improve student learning and satisfaction in Saudi Arabia? | Excluded | Unable to extract or calculate available data |
| 115 | Tyler (2016) | Flipping the CS1 and CS2 Classrooms in Central Asia | Excluded | Records excluded (not pertinent) |
| 116 | Strode (2024) | Exercising Empathy in the Sport Management Classroom: The True Flipped Classroom | Excluded | Subject matter does not fit |
| 117 | Choi (2024) | Comparison of learning effects between hybrid flipped learning and flipped learning | Excluded | Records excluded (not pertinent) |
| 118 | Midtiby (2017) | STUDENTS' BENEFIT FROM VIDEO WITH INTERACTIVE QUIZZES IN A FIRST-YEAR CALCULUS COURSE | Excluded | Records excluded (not pertinent) |
| 119 | Müller (2020) | Technology-supported management education: a systematic review of antecedents of learning effectiveness | Excluded | Review |
| 120 | Silva-López (2017) | A FLIPPED LEARNING APPROACH USING SOCIAL MEDIA. STUDY CASE: ELEMENTAL PROGRAMMING IN HIGHER EDUCATION | Excluded | Records excluded (not pertinent) |
| 121 | Kaulitz (2020) | Digital practical course of otorhinolaryngology and examination technique "to go" | Excluded | Records excluded (not pertinent) |
| 122 | Wang (2022) | Effects of Blended Learning in Physical Education among University Students: A Systematic Review | Excluded | Review |
| 123 | Luster-Teasley (2014) | NSF TUES: Transforming Undergraduate Environmental Engineering Laboratories for Sustainable Engineering using the Case Studies in the Sciences Instructional Method | Excluded | Records excluded (not pertinent) |
| 124 | Kolil (2024) | Virtual labs in chemistry education: A novel approach for increasing student's laboratory educational consciousness and skills | Excluded | Records excluded (not pertinent) |
| 125 | Castillo (2024) | Student Satisfaction with the Implementation of Flipped Class-room Methodology in the Database Applications Course | Excluded | Records excluded (not pertinent) |
| 126 | Zhang (2018) | The Design and Evaluation of Mobile App-Supported Basketball Teaching: A Blended Learning Approach | Excluded | Unable to extract or calculate available data |
| 127 | Matos-Bastidas (2020) | Differences in Knowledge Acquisition, Perceived Engagement and Self-Efficacy in Latino Promotores Delivering the Heart Disease Prevention Program Su Corazόn, Su Vida | Excluded | Records excluded (not pertinent) |
| 128 | Arrizabalaga-Larrañaga (2020) | TEAM-BASED LEARNING STRATEGY TO IMPROVE MASS SPECTRAL INTERPRETATION ON POST-GRADUATE STUDENTS | Excluded | Records excluded (not pertinent) |
| 129 | Shih (2019) | Exploring the role of university students' online self-regulated learning in the flipped classroom: a structural equation model | Excluded | Records excluded (not pertinent) |
| 130 | Yoon (2024) | An Exploratory Study on the Effects of Flipped Learning and Experiential Learning Theory in Physical Educatio | Excluded | Subject matter does not fit |
| 131 | Fang (2022) | Effectiveness Evaluation of Physical Education Flipped Classroom Teaching Based on Knowledge Construction | Excluded | Subject matter does not fit |
| 132 | Tsai (2021) | Impacts of AIOT Implementation Course on the Learning Outcomes of Senior High School Students | Excluded | Records excluded (not pertinent) |
| 133 | Ae (2021) | Exploring the Design of Artificial Intelligence Convergence Liberal Arts Curriculum Based on Flipped Learning and Maker Education: Focusing on Learner Needs Assessment | Excluded | Records excluded (not pertinent) |
| 134 | McLaughlin (2013) | Pharmacy Student Engagement, Performance, and Perception in a Flipped Satellite Classroom | Excluded | Records excluded (not pertinent) |
| 135 | Crawford (2020) | Implementation of the Flipped Classroom to Improve Student Engagement, Academic Success, and Overall Course Satisfaction | Excluded | Records excluded (not pertinent) |
| 136 | Lin (2019) | Research trends of flipped classroom studies for medical courses: a review of journal publications from 2008 to 2017 based on the technology-enhanced learning model | Excluded | Records excluded (not pertinent) |
| 137 | 하명정 (2017) | Using the Inverted Classroom to Teach Writing: A Preliminary Study | Excluded | Records excluded (not pertinent) |
| 138 | Portela (2020) | TechTeach-An Innovative Method to Increase the Students Engagement at Classrooms | Excluded | Records excluded (not pertinent) |
| 139 | Khapre (2021) | Effectiveness of Integrated Google Classroom, Reciprocal Peer Teaching and Flipped Classroom on Learning Outcomes of Research Methodology: A Natural Experiment | Excluded | Records excluded (not pertinent) |
| 140 | Maness (2023) | Maintaining informatics training learning outcomes with a COVID-19 era shift to a fully online flipped course. | Excluded | Records excluded (not pertinent) |
| 141 | Tremonti (2017) | Flipped Classroom Teaching Methods in Medical Education | Excluded | Records excluded (not pertinent) |
| 142 | Lee (2023) | Enhancing Self-Efficacy of Multi-Disciplinary Undergraduates in Arts and Physical Education: A Case Study of Flipped Learning-based Integrative PBL | Excluded | Unable to extract or calculate available data |
| 143 | Desaulniers (2021) | Flipping the Classroom to Teach Undergraduate Physiology during the COVID-19 Pandemic | Excluded | Records excluded (not pertinent) |
| 144 | Gómez (2023) | Analysis of a forced blended-learning program in social sciences higher education during the COVID-19 post-pandemic | Excluded | Records excluded (not pertinent) |
| 145 | Kim (2022) | The Relationship Between Digital Literacy and Learning Effect of Online Classes on College Students majoring in Physical Education | Excluded | Records excluded (not pertinent) |
| 146 | Banks (2021) | An Active Approach to Teaching Central Dogma | Excluded | Records excluded (not pertinent) |
| 147 | Wong (2014) | Pharmacy Students' Performance and Perceptions in a Flipped Teaching Pilot on Cardiac Arrhythmias | Excluded | Records excluded (not pertinent) |
| 148 | García (2015) | THE FLIPPED CLASSROOM THROUGH THE SMARTPHONE: EFFECTS OF ITS EXPERIMENTATION IN HIGH SCHOOL PHYSICAL EDUCATION | Excluded | Subject matter does not fit |
| 149 | Sibia (2022) | The Positive Effects of using Reflective Prompts in a Database Course | Excluded | Records excluded (not pertinent) |
| 150 | Chen (2024) | Information Security Construction of SPOC: Path Selection for Japanese Information Acquisition | Excluded | Records excluded (not pertinent) |
| 151 | Koponen (2019) | The Flipped Classroom Approach for Teaching Cross-cultural Communication to Millennials | Excluded | Records excluded (not pertinent) |
| 152 | Molleda (2019) | A FLIPPED LEARNING EXPERIENCE IN EMBEDDED SYSTEMS IN MASTER DEGREES IN COMPUTER ENGINEERING | Excluded | Records excluded (not pertinent) |
| 153 | Wong (2014) | Pharmacy students' performance and perceptions in a flipped teaching pilot on cardiac arrhythmias. | Excluded | Records excluded (not pertinent) |
| 154 | Jung (2020) | A Study on the Development of Computational Thinking of Scientifically Gifted Children in Physical Computing Classes using a Flipped Learning Strategy | Excluded | Records excluded (not pertinent) |
| 155 | Nunez (2024) | Pharmacology of Sedating and Anesthetic Agents: A Case-Based Flipped Classroom Exercise for Preclinical Medical Students. | Excluded | Records excluded (not pertinent) |
| 156 | Muñoz-Merino (2017) | Flipping the Classroom to Improve Learning With MOOCs Technology | Excluded | Records excluded (not pertinent) |
| 157 | Winfield (2015) | COMMUNITY-BASED INTERACTIVE ENGAGEMENT IN AN ORGANIC CHEMISTRY COURSE | Excluded | Records excluded (not pertinent) |
| 158 | Troufanova (2018) | A FLIPPED CLASSROOM APPROACH TO DEVELOPING TERMINOLOGICAL COMPETENCE IN ENGLISH FOR SPECIFIC PURPOSES | Excluded | Records excluded (not pertinent) |
| 159 | Stenson (2022) | Implementing Ungrading in Undergraduate Exercise Physiology | Excluded | Subject matter does not fit |
| 160 | Kuan (2024) | Students' engagement in online flipped Mandarin as a foreign language learning | Excluded | Records excluded (not pertinent) |
| 161 | Xu (2021) | Analysis of Effectiveness and Performance Prediction of Sports Flipped Classroom Teaching Based on Neural Networks | Excluded | Subject matter does not fit |
| 162 | Mayer (2023) | Introduction to Medical Statistics Software Using the Flipped Classroom: A Pilot Study | Excluded | Records excluded (not pertinent) |
| 163 | Canizales (2020) | Learning style and class environment: situations preceding pedagogical innovation in Sports Science students | Excluded | Subject matter does not fit |
| 164 | MINKYU (2022) | A Study on Applying Flipped Learning in Sports Science Technology Policy Convergence Classes: Focusing on the Conversion of Humanities and Fourth Industrial Revolution Technologies based on Sports Principles, and Its Systematic Class Design and Operation | Excluded | Subject matter does not fit |
| 165 | Alharbi (2023) | A Systematic Literature Review on AI Algorithms and Techniques Adopted by e-Learning Platforms for and Emotional States | Excluded | Review |
| 166 | Henderson (2017) | Introduction of a Nurse Clinical Leadership Simulation Board Game: Wardopoly Undergraduate Nurse Evaluation of Impact | Excluded | Records excluded (not pertinent) |
| 167 | Artal-Sevil (2018) | QR-CODES AS A LEARNING TOOL IN ADVANCED DEGREES | Excluded | Records excluded (not pertinent) |
| 168 | Khanittanuphong (2022) | The impact of the transition from flipped classroom to online lectures on learning outcomes and student satisfaction in a rehabilitation medicine clerkship during the COVID-19 pandemic | Excluded | Records excluded (not pertinent) |
| 169 | 안미영 (2017) | Korean Culture Education through Genre Paintings ― flipped-learning-based instruction | Excluded | Records excluded (not pertinent) |
| 170 | Leu (2012) | NUE: Flipping Learning Models to Illuminate Nanomanufacturing and Nanomaterials for Photovoltaics | Excluded | Records excluded (not pertinent) |
| 171 | Pedreño-Rojas (2020) | Innovative proposal in the Official Master in Diagnosis and Repair of Buildings | Excluded | Records excluded (not pertinent) |
| 172 | Haack (2021) | Remote Laboratory for Activating Lecture Hall Teaching and Distance Learning | Excluded | Records excluded (not pertinent) |
| 173 | Williams (2018) | Creation and implementation of a flipped jigsaw activity to stimulate interest in biochemistry among medical students | Excluded | Records excluded (not pertinent) |
| 174 | Huba (2018) | Flipping the Courses on Automatic Control: Why and How | Excluded | Records excluded (not pertinent) |
| 175 | Campos (2021) | The effectiveness of the Online Flipped Classroom as a didactic strategy for distance education in higher education during the COVID19 quarantine: A case study | Excluded | Records excluded (not pertinent) |
| 176 | Zheng (2022) | Flipped Classroom Approach Used in the Training of Mass Casualty Triage for Medical Undergraduate Students | Excluded | Records excluded (not pertinent) |
| 177 | Feldmann (2023) | Above all: Motivation - some parts of our Maths for Engineers teaching concept | Excluded | Records excluded (not pertinent) |
| 178 | Liu (2021) | An online course mode based on microlecture videos: Using CAD geometric modeling course as an example | Excluded | Records excluded (not pertinent) |
| 179 | Artal-Sevil (2020) | ARE THE NEW METHODOLOGIES IN HIGHER EDUCATION SO EFFECTIVE? DEATH HAD A PRICE | Excluded | Records excluded (not pertinent) |
| 180 | Dong-won (2024) | The Impact of FL (Flipped Learning) Applied Classes on Class Participation and Class Satisfaction of Prospective Special Physical Education Teachers | Excluded | Records excluded (not pertinent) |
| 181 | Bennett (2024) | The learning styles of Australian exercise science and clinical exercise physiology students | Excluded | Subject matter does not fit |
| 182 | Yıldız (2024) | The Effects of Technique-Focused, Play Practice and Flipped Learning Models on Football Game Performance, Tactical Knowledge and Situational Interast of Middle School Students | Excluded | Records excluded (not pertinent) |
| 183 | Ramírez-Donoso (2023) | Fostering the use of online learning resources: results of using a mobile collaboration tool based on gamification in a blended course | Excluded | Records excluded (not pertinent) |
| 184 | López (2017) | FLIPPING THE CLASSROOM BY VIDEO IN MATHEMATICS FOR GEOLOGICAL SCIENCES | Excluded | Records excluded (not pertinent) |
| 185 | Dæhli (2017) | USING BLENDED LEARNING TO DIFFERENTIATE THE LEARNING PROCESS IN A COMPUTER PROGRAMMING COURSE | Excluded | Records excluded (not pertinent) |
| 186 | Cano-Martínez (2019) | EMOTIONAL EDUCATION AS A CROSS CURRICULAR SUBJECT IN SECONDARY SCHOOL: DIDACTIC APPROACHES BASED IN CONFLUENT EDUCATION AND ENHANCED BY GAMIFICATION AND TECHNOLOGY | Excluded | Records excluded (not pertinent) |
| 187 | Campos-Mesa (2022) | Augmented Reality and the Flipped Classroom-A Comparative Analysis of University Student Motivation in Semi-Presence-Based Education Due to COVID-19: A Pilot Study | Excluded | Records excluded (not pertinent) |
| 188 | Agurruza (2018) | THE GAME OF THE CITY | Excluded | Records excluded (not pertinent) |
| 189 | Buil (2020) | "Post-it mapping": analogical disruption in the classroom | Excluded | Records excluded (not pertinent) |
| 190 | Goolsarran (2018) | Effectiveness of an interprofessional patient safety team-based learning simulation experience on healthcare professional trainees | Excluded | Records excluded (not pertinent) |
| 191 | Knoke (2024) | Health promotion in physical education through digital media: a systematic literature review | Excluded | Review |
| 192 | Lee (2024) | Incorporation of Flipped Learning and Problem-Based Learning Methods for the Numerical Analysis Course Design | Excluded | Records excluded (not pertinent) |
| 193 | Delgado (2014) | DEVELOPING TEACHING-LEARNING STRATEGIES TO IMPROVE STUDENTS' UNDERSTANDING, AUTONOMY AND MOTIVATION | Excluded | Records excluded (not pertinent) |
| 194 | Dushyanthen (2025) | Evaluation of an Interdisciplinary Educational Program to Foster Learning Health Systems: Education Evaluation | Excluded | Records excluded (not pertinent) |
| 195 | Young (2016) | Teaching Methods and How People Learn New Things, a Study Based on the Racket Programming Language | Excluded | Records excluded (not pertinent) |
| 196 | Bores-García (2024) | Educational Research on the Use of Virtual Reality Combined with a Practice Teaching Style in Physical Education: A Qualitative Study from the Perspective of Researchers | Excluded | Records excluded (not pertinent) |
| 197 | Abdool (2017) | DataRPG: Improving student motivation in data science through gaming elements | Excluded | Records excluded (not pertinent) |
| 198 | Kaye (2023) | Research Initiation: Improving engineering mechanics self-efficacy by focusing on abstracting the physical world as a precursor to analysis | Excluded | Records excluded (not pertinent) |
| 199 | Crome (2021) | Application of the inverted classroom model in the teaching module "new classification of periodontal and peri-implant diseases and conditions" during the COVID-19 pandemic | Excluded | Records excluded (not pertinent) |
| 200 | Oddsson (2017) | Flipped Classroom Improves the Student's Exam Performance in a First Year Engineering Course | Excluded | Records excluded (not pertinent) |
| 201 | 손준구 (2018) | The Possibility of Self-Directed Learning in Elementary Physical Education Classes | Excluded | Subject matter does not fit |
| 202 | Murnane (2019) | A Classroom Activity to Increase Student Pharmacists Confidence in Dealing with the Opioid Epidemic | Excluded | Records excluded (not pertinent) |
| 203 | Chan (2024) | Use of abdominal thrusts is associated with improved rates of successful emesis induction in dogs | Excluded | Records excluded (not pertinent) |
| 204 | Suire (2024) | Feasibility of the flipped classroom approach for health education in a clinical weight loss program. | Excluded | Records excluded (not pertinent) |
| 205 | Parras (2015) | USE OF WRITTEN TESTS BEFORE/AFTER LECTURES TO IMPROVE COMPREHENSIVE READING BY USING COMPLEMENTARY SOFTWARE | Excluded | Records excluded (not pertinent) |
| 206 | 손승혁 (2024) | Consideration of Ways to Utilize Flipped Learning-based EdTech for Secondary Singing Education | Excluded | Records excluded (not pertinent) |
| 207 | Lakhtakia (2022) | Assessment as Learning in Medical Education: Feasibility and Perceived Impact of Student-Generated Formative Assessments | Excluded | Records excluded (not pertinent) |
| 208 | Everett (2014) | A Hybrid Flipped First Year Engineering Course | Excluded | Records excluded (not pertinent) |
| 209 | O'Leary (2017) | Innovative Pedagogical Approaches to a Capstone Laboratory Course in Cyber Operations | Excluded | Records excluded (not pertinent) |
| 210 | Udroiu (2018) | MODERN LEARNING METHODS USING IN CYBERSECURITY ADULT EDUCATION | Excluded | Records excluded (not pertinent) |
| 211 | Biroli (2022) | Neurological examination lab during SARS-CoV-2 pandemic: an experience from Humanitas University, Milan, Italy | Excluded | Records excluded (not pertinent) |
| 212 | Hayward (016) | Rheumapalooza Update: Applying a Flipped Classroom Instructional Model to an Intensive Rheumatology Curriculum for Second Year Medical Students | Excluded | Records excluded (not pertinent) |
| 213 | Janfeshan (2024) | Students Perceptions in Linear Control Laboratory with TEL | Excluded | Records excluded (not pertinent) |
| 214 | Chang (2020) | Learning With Jigsaw Puzzle and Augmented Reality-A Case Study of Biology | Excluded | Records excluded (not pertinent) |
| 215 | Beason-Abmayr (2019) | Does Implementation of Flipped Teaching Combined with Retrieval Practice Enhance Student Engagement in Class Discussions? | Excluded | Records excluded (not pertinent) |
| 216 | Gastardo (2016) | ENHANCING UNDERGRADUATE MATHEMATICS LEARNING EXPERIENCE: FLIPPED LESSONS WITH INTEGRATED VIDEO | Excluded | Records excluded (not pertinent) |
| 217 | Fragkaki (2021) | Distance Higher Education Learning and Professional Pedagogy: Training the Trainers | Excluded | Records excluded (not pertinent) |
| 218 | Stiehm (2016) | BLENDED LEARNING THROUGH INTEGRATING LEGO MINDSTORMS NXT ROBOTS IN ENGINEERING EDUCATION | Excluded | Records excluded (not pertinent) |
| 219 | Elmer (2016) | Blended learning within an undergraduate exercise physiology laboratory | Excluded | Records excluded (not pertinent) |
| 220 | Kolveková (2017) | Changes in human resources in education sector and its institutions in Slovak Republic | Excluded | Records excluded (not pertinent) |
| 221 | Olivier (2020) | Digital technologies in undergraduate and postgraduate education in occupational therapy and physiotherapy: a scoping review | Excluded | Records excluded (not pertinent) |
| 222 | Yang (2021) | Effectiveness of neonatal emergency nursing education through simulation training: Flipped learning based on Tanner's Clinical Judgement Model | Excluded | Records excluded (not pertinent) |
| 223 | Messina (2016) | MICRO-DESIGN IN SPECIAL EDUCATION | Excluded | Records excluded (not pertinent) |
| 224 | Franco (2022) | The André Cruz de Carvalho Active Learning Classroom: Building New Learning Spaces | Excluded | Records excluded (not pertinent) |
| 225 | MinKyung (2023) | Development of Software Education Program for Fostering Computational Thinking in the Era of the 4th Industrial Revolution | Excluded | Records excluded (not pertinent) |
| 226 | Won (2023) | Machine Learning Driven Synthesis of Clock Gating | Excluded | Records excluded (not pertinent) |
| 227 | Won (2023) | Machine Learning Driven Synthesis of Clock Gating | Excluded | Records excluded (not pertinent) |
| 228 | da Silva Pinto (2024) | The Geographical Exploration of Online News, in Geography Teaching - Case Study with 8th Year Students at Escola Básica e Secundária de Canelas | Excluded | Records excluded (not pertinent) |
| 229 | Adesso (2019) | Light the world and change its color: a case study in Italian secondary school using IBSE methodology | Excluded | Records excluded (not pertinent) |
| 230 | de Lima (2022) | Hybrid education in healthcare education : a systematic review | Excluded | Records excluded (not pertinent) |
| 231 | Luís (2018) | Self-assessment as a Learning Instrument in a Flipped University Course | Excluded | Records excluded (not pertinent) |
| 232 | Backlund (2020) | A Comprehensive Course for Teaching Emergency Cricothyrotomy. | Excluded | Records excluded (not pertinent) |
| 233 | Ferguson (2017) | The integration and evaluation of a social-media facilitated journal club to enhance the student learning experience of evidence-based practice: A case study | Excluded | Records excluded (not pertinent) |
| 234 | Plastow (2018) | Flipping and Drawing to Enhance Student Engagement and Learning in Dental Anatomy | Excluded | Records excluded (not pertinent) |
| 235 | Swift (2014) | A Partial Flip, A Whole Transformation: Redesigning Sophomore Circuits | Excluded | Records excluded (not pertinent) |
| 236 | Wiley (2018) | Human Papillomavirus: From Basic Science to Clinical Management for Preclinical Medical Students. | Excluded | Records excluded (not pertinent) |
| 237 | Walz (2024) | Actively Teaching Active Teaching Techniques. | Excluded | Records excluded (not pertinent) |
| 238 | Xie (2023) | A Comparative Study of Direct Instruction and Flipped Classroom Teaching Methods on College Students' Learning Performance and Self-Efficacy in Physical Education | Excluded | Records excluded (not pertinent) |
| 239 | Jia (2022) | Experimental study of flipped classroom teaching in the flower ball cheerleading option class of vocational colleges and universities | Excluded | Records excluded (not pertinent) |
| 240 | Wu (2021) | Research on the Application of Flipped Classroom in College Sports Volleyball Teaching in Colleges and Universities | Excluded | Records excluded (not pertinent) |
| 241 | Zeng (2021) | Application of flipping tracking teaching method in Aerobics compulsory course Experimental research in Teaching | Excluded | Records excluded (not pertinent) |
| 242 | Zou (2021) | Survey on the Current Status of Online Teaching of Public Physical Education Courses in Some Universities in Shanghai during the Epidemic and Countermeasures Research | Excluded | Records excluded (not pertinent) |
| 243 | Zhou (2020) | Practice Research and Analysis of Calisthenics Online Teaching Based on Flipped Classroom Model | Excluded | Records excluded (not pertinent) |
| 244 | Zhao (2021) | Study on the Outcome-Oriented Influencing Factors of Student Satisfaction in Modern Educational Technology Course | Excluded | Records excluded (not pertinent) |
| 245 | Jiang (2020) | An Empirical Study on the Impact of Flipped Classroom Teaching on College Students' Self-directed Learning Ability in Physical Education - A Case Study of Public Wushu Class in Xi'an University of Posts and Telecommunications | Excluded | Records excluded (not pertinent) |
| 246 | Wang (2019) | Study on the Learning Autonomy Effect of Flip Classroom Teaching in College Volleyball Public Elective Course | Excluded | Records excluded (not pertinent) |
| 247 | Zhang (2017) | A Study of the Effectiveness of Flipped Classrooms in Physical Education Courses | Excluded | Records excluded (not pertinent) |
| 248 | Li (2015) | Opportunity and Challenge——the Enlightenment of the Flipped Classroom Approach on Collegiate Physical Education | Excluded | Records excluded (not pertinent) |
| 249 | Li (2015) | The application and learning satisfaction of blended learning in the psychology of physical education | Excluded | Records excluded (not pertinent) |
| 250 | Ding (2020) | An experimental study on theflipped classroom of physicaleducation in Higher VocationalColleges by using the APP of blueink cloud class——Take Yantai EngineeringVocational and Technical Collegeas an example | Excluded | Records excluded (not pertinent) |
| 251 | Li (2020) | Research on the application of flipped classroom in calisthenicsteaching in universities from the perspective of core literacy | Excluded | Records excluded (not pertinent) |
| 252 | Xu (2019) | Study on the effect of “flipped classroom” teaching mode in cheerleading option class | Excluded | Records excluded (not pertinent) |
| 253 | Du (2024) | An Experimental Study of the Flipped Classroom in Teaching Alpine Skiing | Excluded | Records excluded (not pertinent) |
| 254 | Yuan (2024) | Design and Experimental Researe of SPOC Hybrid Teaching Based on Learning Baeagemient Theory in Basketball Compulsory Courses in Physical Education Colleges | Excluded | Records excluded (not pertinent) |
| 255 | Song (2024) | Design and application of SPOC teaching model Take the course "Computer Network" as an example | Excluded | Records excluded (not pertinent) |
| 256 | Li (2023) | A Study of Building College Sports Flipped Classroom Based on ARCS Model | Excluded | Records excluded (not pertinent) |
| 257 | Fan (2023) | Dissertation Submitted to Guangzhou Sport University for Master Degree | Excluded | Records excluded (not pertinent) |
| 258 | Song (2023) | Research on the Application of "PAD Class" Teaching Mode in High School Basketball | Excluded | Records excluded (not pertinent) |
| 259 | Zhang (2023) | The research on the application of "Counterpoint Classroom"model in back style High Jump teaching | Excluded | Records excluded (not pertinent) |
| 260 | Yang (2023) | The Design and Practice of Blended Teaching Mode based on Community of Inquiry Theory in Table Tennis Umpire Course | Excluded | Records excluded (not pertinent) |
| 261 | Lin (2023) | Application research of Flipped Classroom based on "Douyin Short Video app" in Table tennis elective course teaching in colleges and universities | Excluded | Records excluded (not pertinent) |
| 262 | Chen (2023) | Experimental Research on TBL Teaching Method Based on WeChat Platform in Aerobics Elective Courses in Ordinary Universities | Excluded | Records excluded (not pertinent) |
| 263 | Li (2023) | SPOC blended pedagogy applied to university badminton teaching——Take Harbin Normal University as an examp | Excluded | Records excluded (not pertinent) |
| 264 | Wan (2023) | Research on the Application Effect of " Flip Classroom " in the Teaching of Air Volleyball Elective Course in Middle School——Taking the Middle School Affiliated to Jiangxi University of Science and Technology as an Example | Excluded | Records excluded (not pertinent) |
| 265 | Peng (2023) | A Study on the Satisfaction of Tennis Class Teaching--Based on the Survey of Tennis Class in Wuhan Sports university | Excluded | Records excluded (not pertinent) |
| 266 | Cai (2022) | An Applied Study of the Use of Smart Classroom Teaching Mode in College Sports Dance Classes | Excluded | Records excluded (not pertinent) |
| 267 | Li (2022) | An Experimental Study on Enhancing the Teaching Quality of Cheerleading in Colleges and Universities by Dividing the Classroom | Excluded | Records excluded (not pertinent) |
| 268 | Wang (2022) | Research on teaching design and  application of general gymnastics courses  for physical education majors under SPOC mode | Excluded | Records excluded (not pertinent) |
| 269 | Ji (2022) | Construction of a System for Evaluating the Quality of Classroom Teaching in Martial Arts Routines under CIPP Mode | Excluded | Records excluded (not pertinent) |
| 270 | Han (2022) | Experimental research on the effect of flipped classroom teaching mode on Badminton Teaching in Senior High School | Excluded | Records excluded (not pertinent) |
| 271 | Xie (2022) | Action Research on the application of "online and offline"mixed teaching method in wushu course teaching in colleges and universities | Excluded | Records excluded (not pertinent) |
| 272 | Liu (2022) | Experimental research on online and offline mixed teaching in Aerobics Teaching of Physical Education Specialty | Excluded | Records excluded (not pertinent) |
| 273 | Yan (2022) | Research on the application effect of flipped Classroom in gymnastics teaching of physical Education Specialty-- A case study of Shenyang University | Excluded | Records excluded (not pertinent) |
| 274 | Gu (2022) | The application of micro lesson in the teaching of cheer leading in universities | Excluded | Records excluded (not pertinent) |
| 275 | Zhou (2022) | Experimental research on "Double points"Teaching Method in football teaching in Higher Vocational College | Excluded | Records excluded (not pertinent) |
| 276 | Ma (2022) | The practical difficulties and relief strategies of online teaching of physical education courses in colleges and universities in Henan Province | Excluded | Records excluded (not pertinent) |
| 277 | Zhang (2021) | Design and effect analysis of hybrid teaching design of college basketball class based on mobile terminal | Excluded | Records excluded (not pertinent) |
| 278 | Xiao (2021) | Research on the influence of flipped classroom teaching mode on physical education teaching effect and physical activity behavior under the Learning Pass platform | Excluded | Records excluded (not pertinent) |
| 279 | Zhang (2021) | Research on Online Teaching Effect of Basketball Special Course in Colleges and Universities of Jilin Province under the Background of "COVID-19 Epidemic" | Excluded | Records excluded (not pertinent) |
| 280 | Liu (2021) | Research on the Application of SPOC based Flipped classroom Teaching Mode in the Elective course of Public Sports aerobics in universities | Excluded | Records excluded (not pertinent) |
| 281 | Zhang (2021) | Reflections on Online Teaching of Volleyball Courses for college Physical Education Majors in Gansu Province under the “COVID-19”Epidemic | Excluded | Records excluded (not pertinent) |
| 282 | Zhao (2021) | Experimental study on the mixed teaching mode of body dance in colleges and universities | Excluded | Records excluded (not pertinent) |
| 283 | Liu (2021) | Research on Practical Innovation of O2OTeaching Mode of Aerobics in Colleges and Universities——Taking Southwest University as an Example | Excluded | Records excluded (not pertinent) |
| 284 | Ji (2021) | Experimental Research on the Influence of Shifting Classroom Teaching Mode on the Learning Effect of College Yoga Elective Courses | Excluded | Records excluded (not pertinent) |
| 285 | Li (2021) | The Application Research of Blengded Learning in Tennis Selective Course in Physical Education Institutes | Excluded | Records excluded (not pertinent) |
| 286 | Liu (2021) | Study on the Effects of the Application of"PAD Class" Teaching Mode in Long Jump | Excluded | Records excluded (not pertinent) |
| 287 | Zhang (2021) | Research on Online Teaching of Physical Education Major in Western Liaoning Universities During the Outbreak of COVID-1 | Excluded | Records excluded (not pertinent) |
| 288 | Gu (2021) | Experimental Study on the Application of SPOC Teaching Mode in the Optional Course of Public Sports Volleyball in Colleges and Universities | Excluded | Records excluded (not pertinent) |
| 289 | Li (2021) | Practical research of flipped classroom teaching mode based on SPOC in sports biomechanics Teaching | Excluded | Records excluded (not pertinent) |
| 290 | Feng (2020) | Research on the Application of Flipping Classroom Teaching in Volleyball Teaching in Vocational Colleges——Take Liaocheng Institute of Technicians as an example | Excluded | Records excluded (not pertinent) |
| 291 | Yang (2020) | Influence of Flipped Classroom Based on MOOC Teaching Model on Satisfaction and Learning Motivation of College Physical Education | Excluded | Records excluded (not pertinent) |
| 292 | Lu (2020) | The Application of Mixed Learning Evaluation in the Teaching of Basketball Subject under the Flipped Classroom Model | Excluded | Unable to extract or calculate available data |
| 293 | Zhao (2020) | Study on the Outcome-Oriented Influencing Factors of Student Satisfaction in Modern Educational Technology Course | Excluded | Records excluded (not pertinent) |
| 294 | Gu (2020) | Research on the application of flipped classroom teaching model in level three football class | Excluded | Records excluded (not pertinent) |
| 295 | Lin (2020) | Dissertation Submitted to Guangzhou Sport University for Master Degree | Excluded | Records excluded (not pertinent) |
| 296 | Jia (2020) | Research on the Application of Intelligent Teaching Platform in Volleyball General Course of Guangzhou Institute of Physical Education | Excluded | Records excluded (not pertinent) |
| 297 | Shen (2020) | Research on the influence of video teaching on the teaching effect of Public Aerobics in Colleges and Universities Based on the concept of Hybrid Teaching | Excluded | Records excluded (not pertinent) |
| 298 | Tian (2020) | The design and application of the new teaching mode of flipped classroom based on SPOC—Take the course of table tennis competition judging theory and method as an example | Excluded | Records excluded (not pertinent) |
| 299 | Ding (2020) | An experimental study on the flipped classroom of physical education in Higher Vocational Colleges by using the APP of blue ink cloud class——Take Yantai Engineering Vocational and Technical College as an example | Excluded | Records excluded (not pertinent) |
| 300 | Dai (2020) | An experimental study of micro class in children's Latin dance teaching based on SPOC teaching mode -- Taking Changsha Chenxi school as an example | Excluded | Records excluded (not pertinent) |
| 301 | Wu (2020) | Experimental research on flipped classroom teaching model in college table tennis elective | Excluded | Records excluded (not pertinent) |
| 302 | Wang (2020) | Analysis of Satisfaction of Flipped Classroom Application in Teaching Elementary School Physical Education Classes | Excluded | Unable to extract or calculate available data |
| 303 | Yin (2019) | Research on the Application of Flipped Classroom Teaching Mode Based on Online Course Platform in University Physical Fitness Course | Excluded | Records excluded (not pertinent) |
| 304 | Luo (2019) | Research on the Construction and Practice of the Teaching Mode of Flip Classroom of Opera in High Vocational Schools Viewing from the Perspective of MOOC | Excluded | Records excluded (not pertinent) |
| 305 | Chen (2019) | Research on the Application of Flipped Classroom Teaching in Sports Marketing | Excluded | Records excluded (not pertinent) |
| 306 | Cui (2019) | Experimental Study on the Application of Flip Classroom Teaching Model in the Public Aerobics Optional Course of Colleges and Universities | Excluded | Records excluded (not pertinent) |
| 307 | Huang (2019) | Study on the implementation of Rain Classroom in “Sports Physiology” | Excluded | Records excluded (not pertinent) |
| 308 | Hua (2019) | Construction and implication of Physical Education Teaching Design in Elementary School as a national quality resource sharing course | Excluded | Records excluded (not pertinent) |
| 309 | Zhang (2019) | A Practical Study of Flipped Classroom in Physical Education Based on Blue Ink Cloud Class Platform --Taking the Teaching of Long Fist in University Physical Education as an Example | Excluded | Records excluded (not pertinent) |
| 310 | Xu (2019) | Research on the Influence of "Flipping Classroom" on the Teaching Effect of Martial arts Classroom in Colleges and Universities—Taking Xi'an Vocational and Technical College as an example | Excluded | Records excluded (not pertinent) |
| 311 | Liu (2019) | Research on the Application of Flipping Classroom to Taijiquan Teaching in Colleges and Universities | Excluded | Records excluded (not pertinent) |
| 312 | Cai (2019) | Preliminary Study on the Application of Flipped Classroom Teaching Mode in Table Tennis Elective Course Based on the Micro-course | Excluded | Unable to extract or calculate available data |
| 313 | Guo (2018) | Application of flipped classroom teaching model in teaching exercise physiology | Excluded | Records excluded (not pertinent) |
| 314 | Chen (2018) | The Application of SPOC in College Physical Education—Take the teaching of public tennis elective course of fujian normal university as an example | Excluded | Records excluded (not pertinent) |
| 315 | Liu (2018) | Experimental Research on the Effect of Flipping Classroom TeachingModel in College Volleyball Optional Course | Excluded | Records excluded (not pertinent) |
| 316 | Zuo (2018) | Research on the Influence of "Flipping Class" Teaching Mode on Junior Middle School Physical Education Teaching Effect | Excluded | Records excluded (not pertinent) |
| 317 | Lu (2018) | Construction and Research on the Teaching Mode of SPOC Flipped Classroom——A Case Study on Professional Basketball Courses in Colleges | Excluded | Unable to extract or calculate available data |
| 318 | Xie (2017) | The Application of Flipped Classroom in the Course of College Aerobics | Excluded | Records excluded (not pertinent) |
| 319 | Wang (2017) | Danceing Cheerleading mixed in common colleges and universities teaching mode of experiment research - in guangxi normal university, for example | Excluded | Records excluded (not pertinent) |

| **S2 Table. Risk of Bias Assessment.** | | | | | | | |
| --- | --- | --- | --- | --- | --- | --- | --- |
| **Study ID** | **Random sequence generation** | **Allocation concealment** | **Blinding of participants and personnel** | **Blinding of outcome assessment** | **Incomplete outcome data** | **Selective reporting** | **Other biases** |
| 1 | Unclear | Low risk | Unclear | Unclear | Low risk | Low risk | Unclear |
| 2 | Low risk | Low risk | Unclear | Low risk | Low risk | Low risk | Low risk |
| 3 | Low risk | Low risk | Unclear | Unclear | Low risk | Low risk | Unclear |
| 4 | Low risk | Low risk | High risk | Unclear | Low risk | Low risk | Low risk |
| 5 | Low risk | Low risk | Unclear | Unclear | Low risk | Low risk | Unclear |
| 6 | Unclear | Low risk | Unclear | Low risk | Low risk | Low risk | Low risk |
| 7 | Low risk | Low risk | Unclear | Unclear | Low risk | Low risk | Low risk |
| 8 | Low risk | Low risk | High risk | Low risk | Low risk | Low risk | Low risk |
| 9 | High risk | High risk | Unclear | Unclear | Low risk | Low risk | Low risk |
| 10 | Low risk | Low risk | Unclear | Unclear | Low risk | Low risk | Unclear |
| 11 | Low risk | Low risk | High risk | High risk | Low risk | Low risk | Low risk |
| 12 | Low risk | Low risk | High risk | Low risk | Low risk | Low risk | Low risk |
| 13 | Low risk | Low risk | Unclear | Low risk | Low risk | Low risk | Low risk |

| **S3 Table. Results of publication bias in meta-analyses.** | | | | | |
| --- | --- | --- | --- | --- | --- |
| **Std_Eff** | **Coefficient** | **Std. err.** | **t** | **P>\|t\|** | **95% CI** |
| **Intrinsic motivation** | | | | | |
| Slope | 0.45 | 0.27 | 1.70 | 0.12 | (-0.15, 1.06) |
| Bias | 0.88 | 1.32 | 0.66 | 0.52 | (-2.11, 3.87) |
| **Self-efficacy** | | | | | |
| Slope | 1.33 | 1.15 | 1.16 | 0.33 | (-2.33, 4.99) |
| Bias | -4.50 | 5.85 | -0.77 | 0.50 | (-23.12, 14.12) |
| **Learning satisfaction** | | | | | |
| Slope | 1.17 | 1.15 | 1.02 | 0.38 | (-2.49, 4.84) |
| Bias | -2.15 | 5.17 | -0.42 | 0.71 | (-18.61, 14.30) |

| **S4 Table. Minimal data set.** | | | | | | | |
| --- | --- | --- | --- | --- | --- | --- | --- |
| **ID** | **Year** | **Research topics** | **Country** | **Populations** | **Class sizes** | **Subjects** | **Effect size**  **(mean±SD)** |
| Osterlie(F) | 2020 | Intrinsic motivation | Norway | Secondary school | >40 | Multi-sport | E: 5.26±1.44  C: 4.62±1.58 |
| Osterlie(M) | 2020 | Intrinsic motivation | Norway | Secondary school | >40 | Multi-sport | E: 5.32±1.27  C: 4.78±1.69 |
| Yip | 2020 | Intrinsic motivation | China | Primary school | >40 | Sprint | E: 3.27±0.75  C: 3.06±0.70 |
| Ferriz-Valero | 2022 | Intrinsic motivation | Spain | Secondary school | >40 | Ball sports | E: 4.40±0.51  C: 3.77±0.83 |
| Ridwan(F) | 2023 | Intrinsic motivation | Indonesia | University | ≤40 | NR | E: 64.7±4.64  C: 60.7±3.52 |
| Ridwan(M) | 2023 | Intrinsic motivation | Indonesia | University | ≤40 | NR | E: 75.1±11.5  C: 68.5±7.09 |
| Ferriz-Valero | 2022 | Intrinsic motivation | Spain | Secondary school | >40 | Ball sports | E: 3.98±0.80  C: 3.64±0.80 |
| Lin | 2021 | Intrinsic motivation | China | University | ≤40 | Ball sports | E: 4.22±0.75  C: 3.95±0.63 |
| Lucena(a) | 2020 | Intrinsic motivation | Spain | Primary school | ≤40 | NR | E: 2.73±1.03  C: 2.00±0.89 |
| Lucena(b) | 2020 | Intrinsic motivation | Spain | Secondary school | ≤40 | NR | E: 2.66±0.92  C: 1.87±0.85 |
| Karaman | 2023 | Intrinsic motivation | Turkiye | Secondary school | ≤40 | Ball sports | E: 4.46±0.55  C: 3.46±0.72 |
| Lin | 2021 | Self-efficacy | China | University | ≤40 | Ball sports | E: 4.25±0.59  C: 4.09±0.60 |
| Lin(a) | 2019 | Self-efficacy | China | University | ≤40 | Dance | E: 3.04±0.74  C: 3.04±0.83 |
| Lin(b) | 2019 | Self-efficacy | China | University | ≤40 | Dance | E: 3.60±0.95  C: 3.04±0.83 |
| Hu | 2018 | Self-efficacy | China | University | >40 | Ball sports | E: 36.28±3.814  C: 35.68±3.056 |
| Li | 2019 | Self-efficacy | China | University | >40 | Ball sports | E: 27.67±3.84  C: 24.10±3.52 |
| Lin(a) | 2019 | Learning satisfaction | China | University | ≤40 | Dance | E: 3.59±0.66  C: 3.29±0.65 |
| Lin(b) | 2019 | Learning satisfaction | China | University | ≤40 | Dance | E: 3.94±0.85  C: 3.29±0.65 |
| Chao(a) | 2021 | Learning satisfaction | China | University | >40 | Dance | E: 18.54±2.54  C: 17.28±3.27 |
| Chao(b) | 2021 | Learning satisfaction | China (foreign student) | University | ≤40 | Dance | E: 18.52±3.06  C: 17.63±3.40 |
| Li | 2018 | Learning satisfaction | China | University | >40 | Ball sports | E: 91.14±7.28  C: 82.46±6.28 |
| a or b: different subgroups in the same study; C: control group; E: experimental group; F: female; SD, standard deviation; M: male; NR, not reported. | | | | | | | |


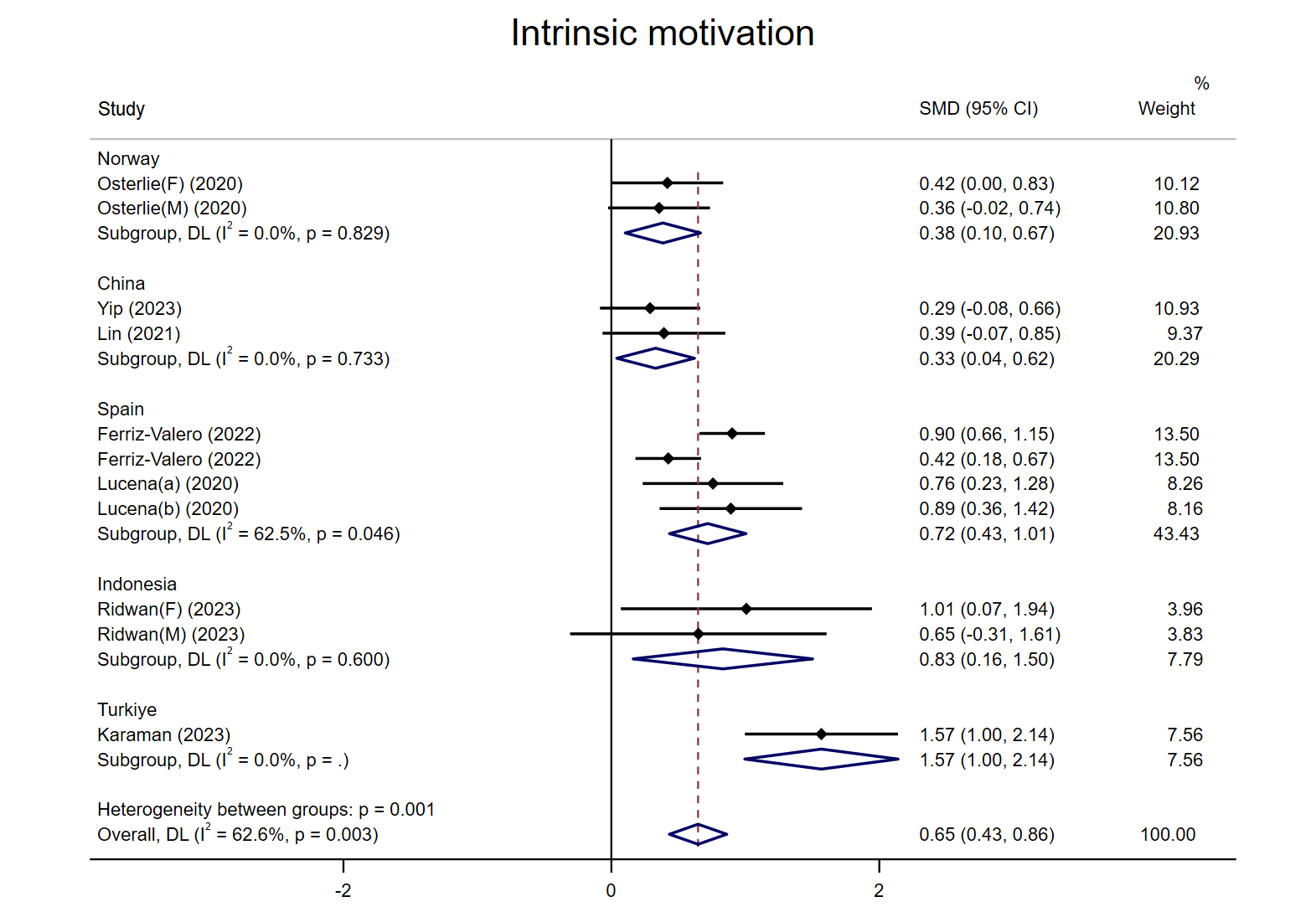


**S1 Fig. The effect of FC versus traditional PE on students' intrinsic motivation by country.**


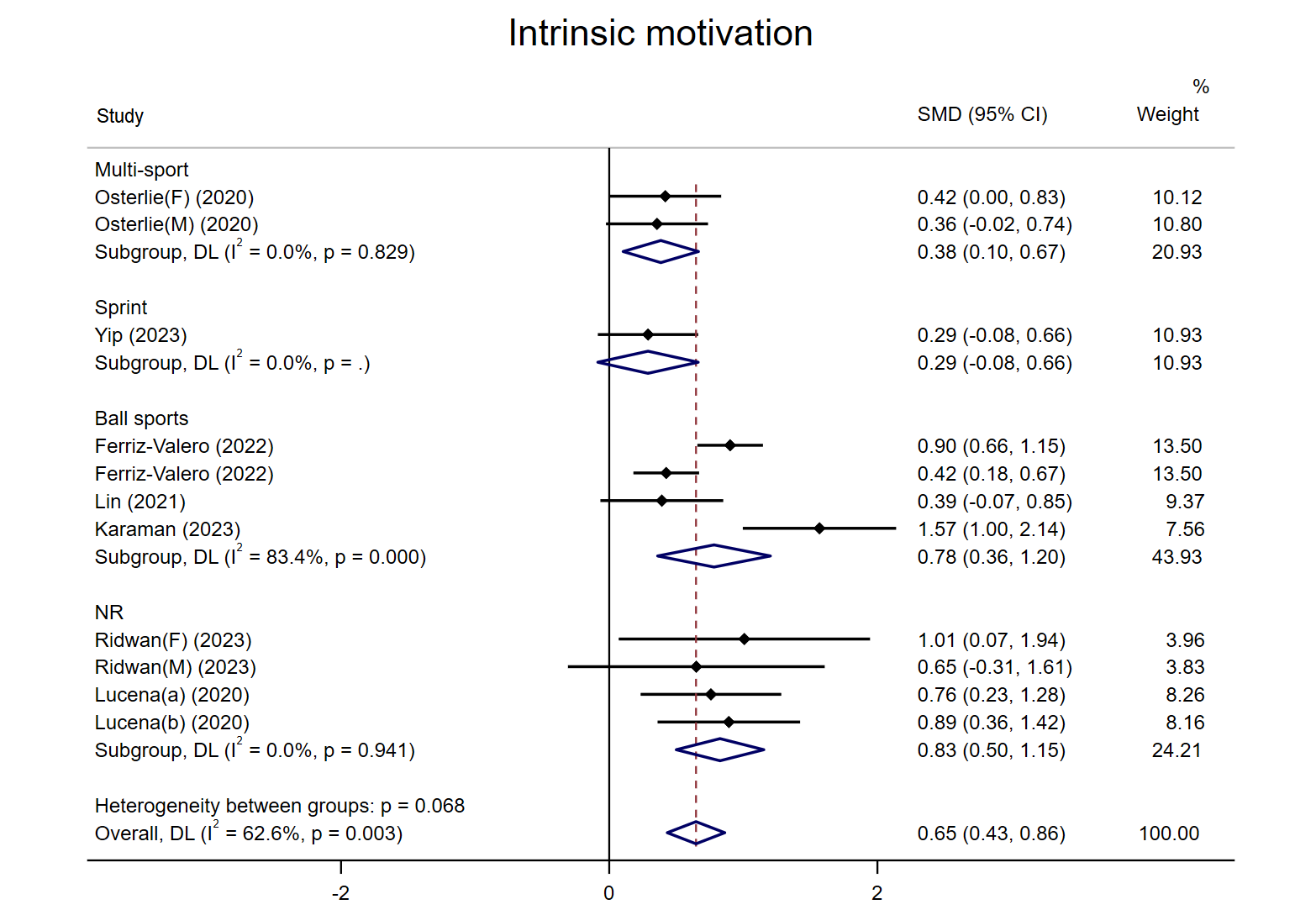


**S2 Fig. The effect of FC versus traditional PE on students' intrinsic motivation by** **PE subject.**


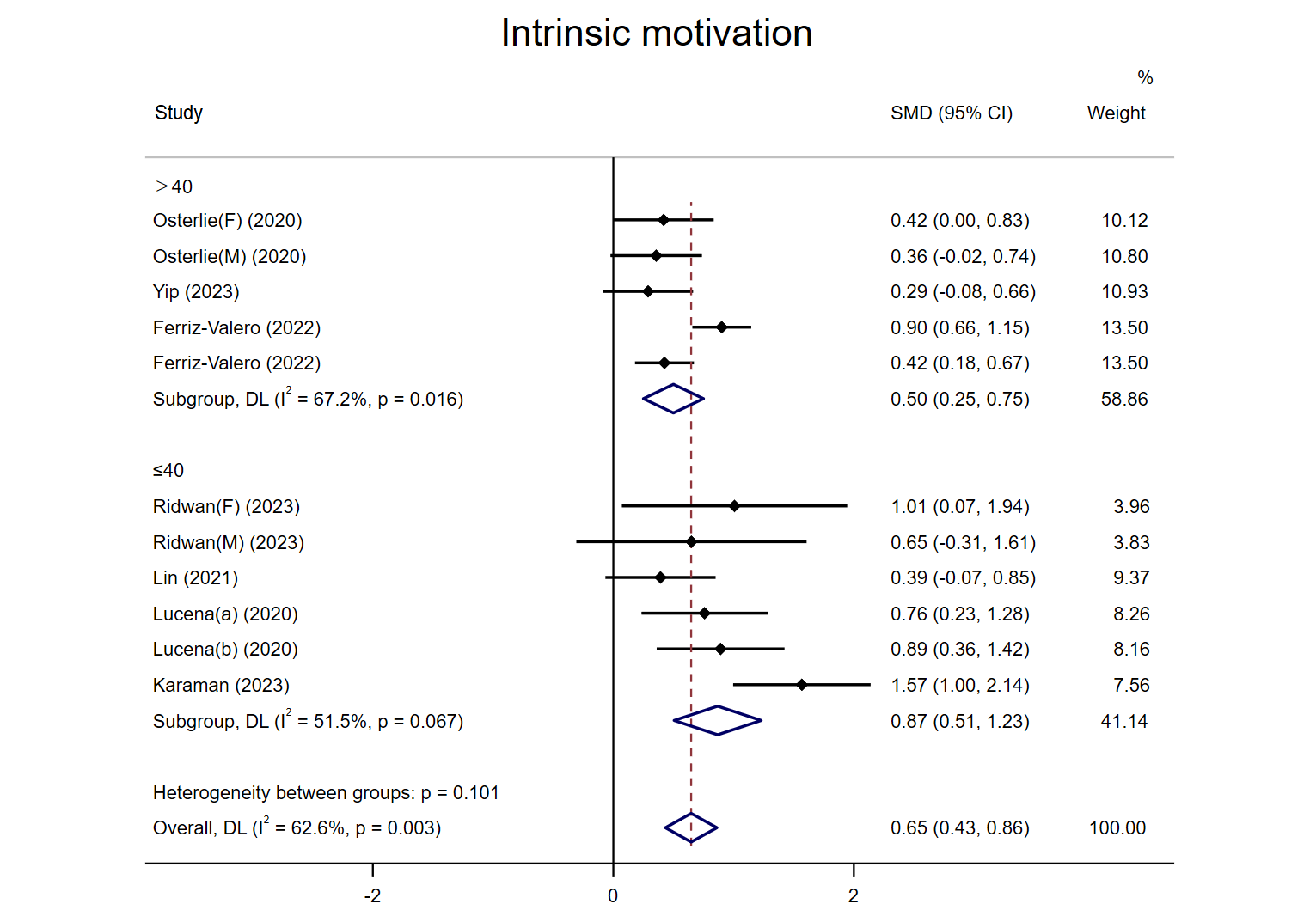


**S3 Fig. The effect of FC versus traditional PE on students' intrinsic motivation by class sizes.**


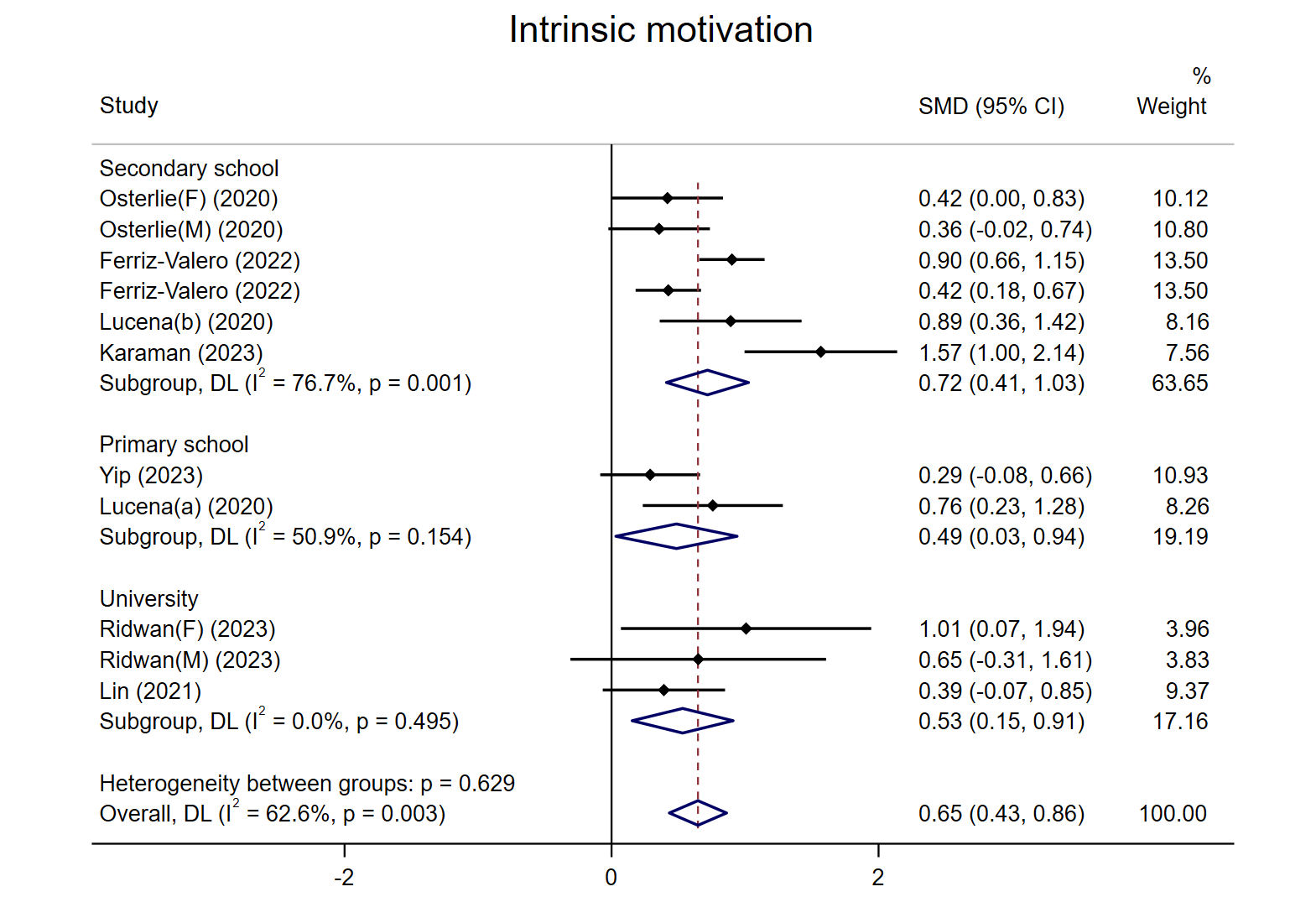


**S4 Fig. The effect of FC versus traditional PE on students' intrinsic motivation by** **population.**





**S5 Fig. Leave-one-out analysis for the association of FC with intrinsic motivation.**


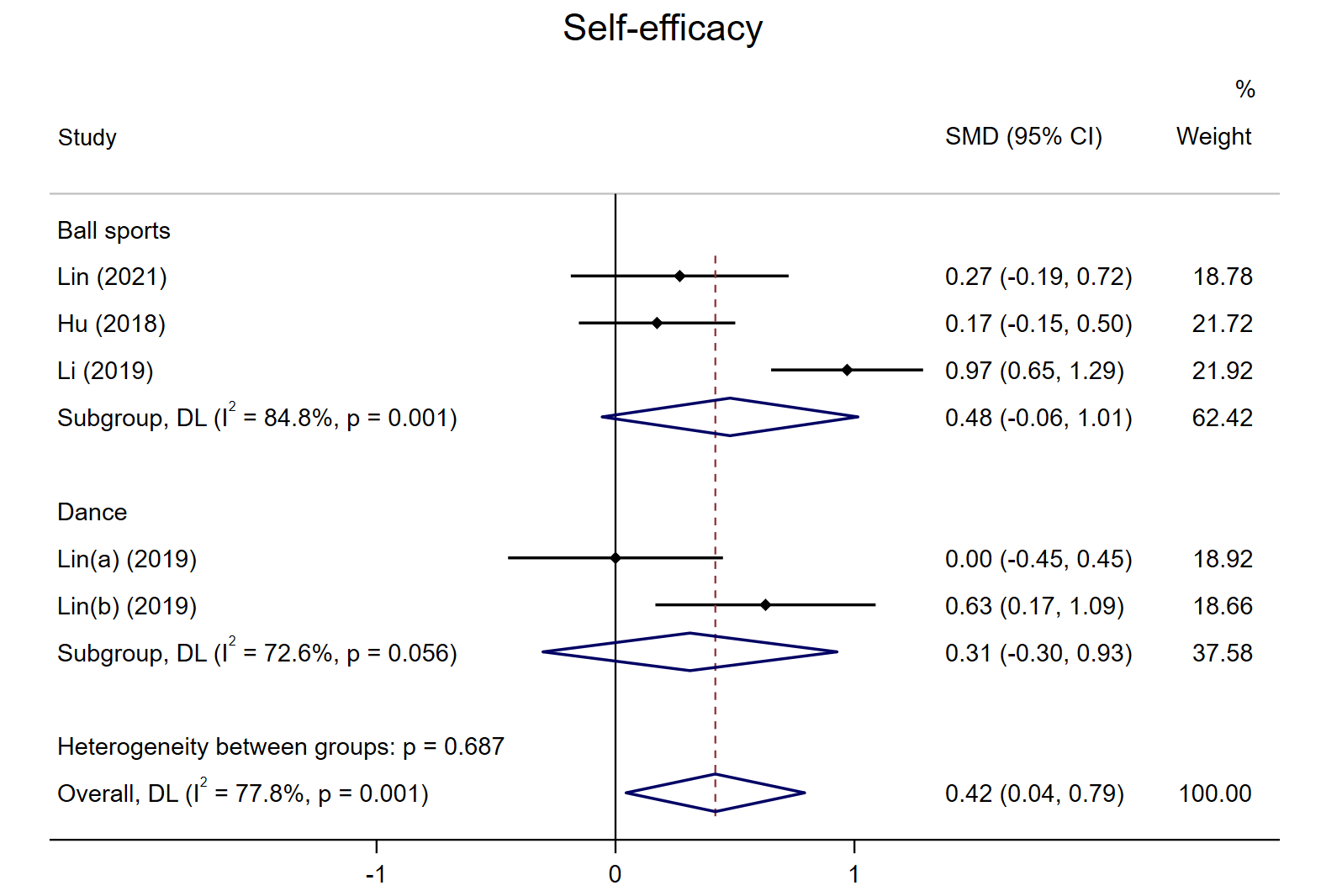


**S6 Fig. The effect of FC versus traditional PE on students' self-efficacy by PE subject.**


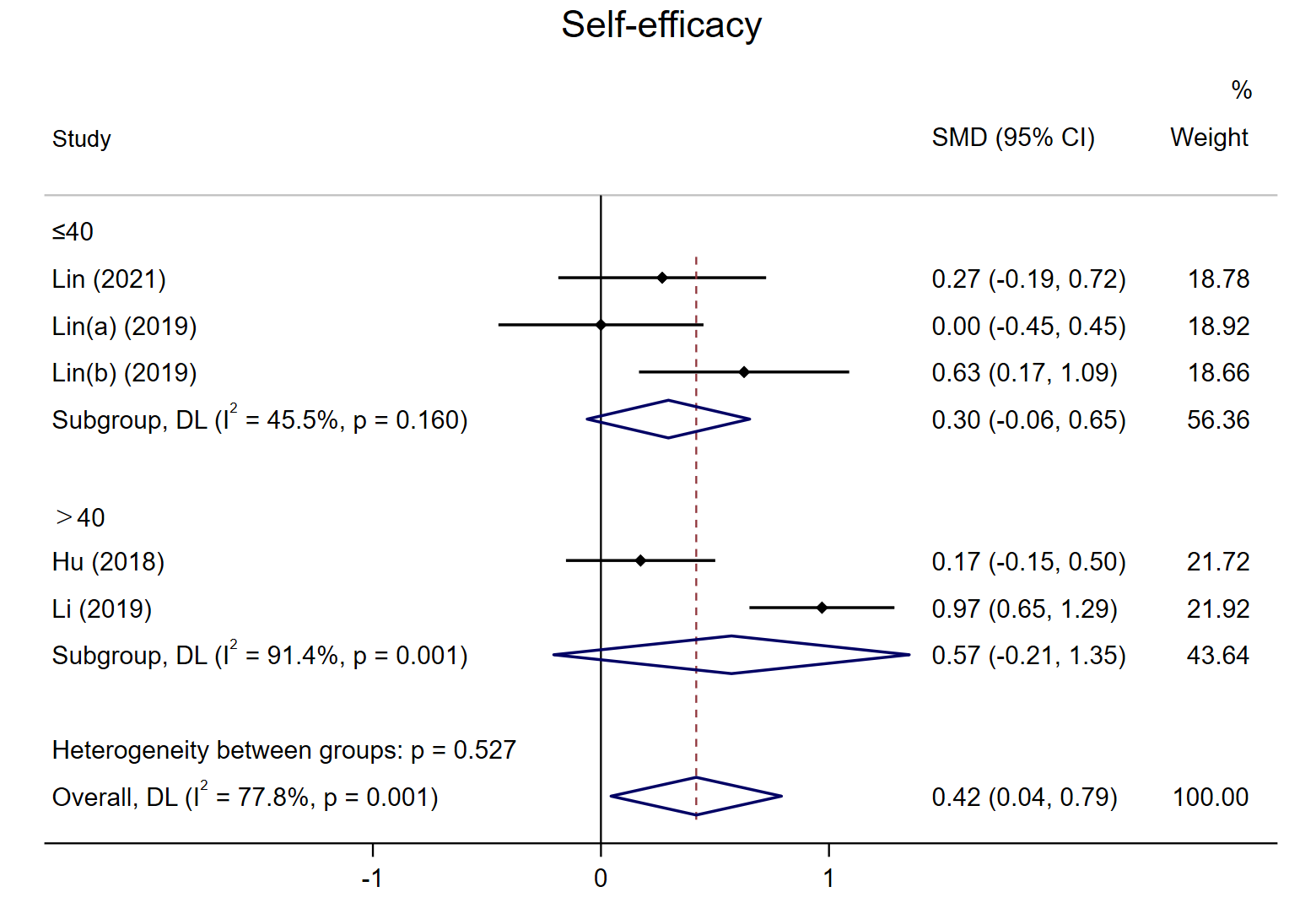


**S7 Fig. The effect of FC versus traditional PE on students' self-efficacy by class sizes.**





**S8 Fig. Leave-one-out analysis for the association of FC with self-efficacy.**


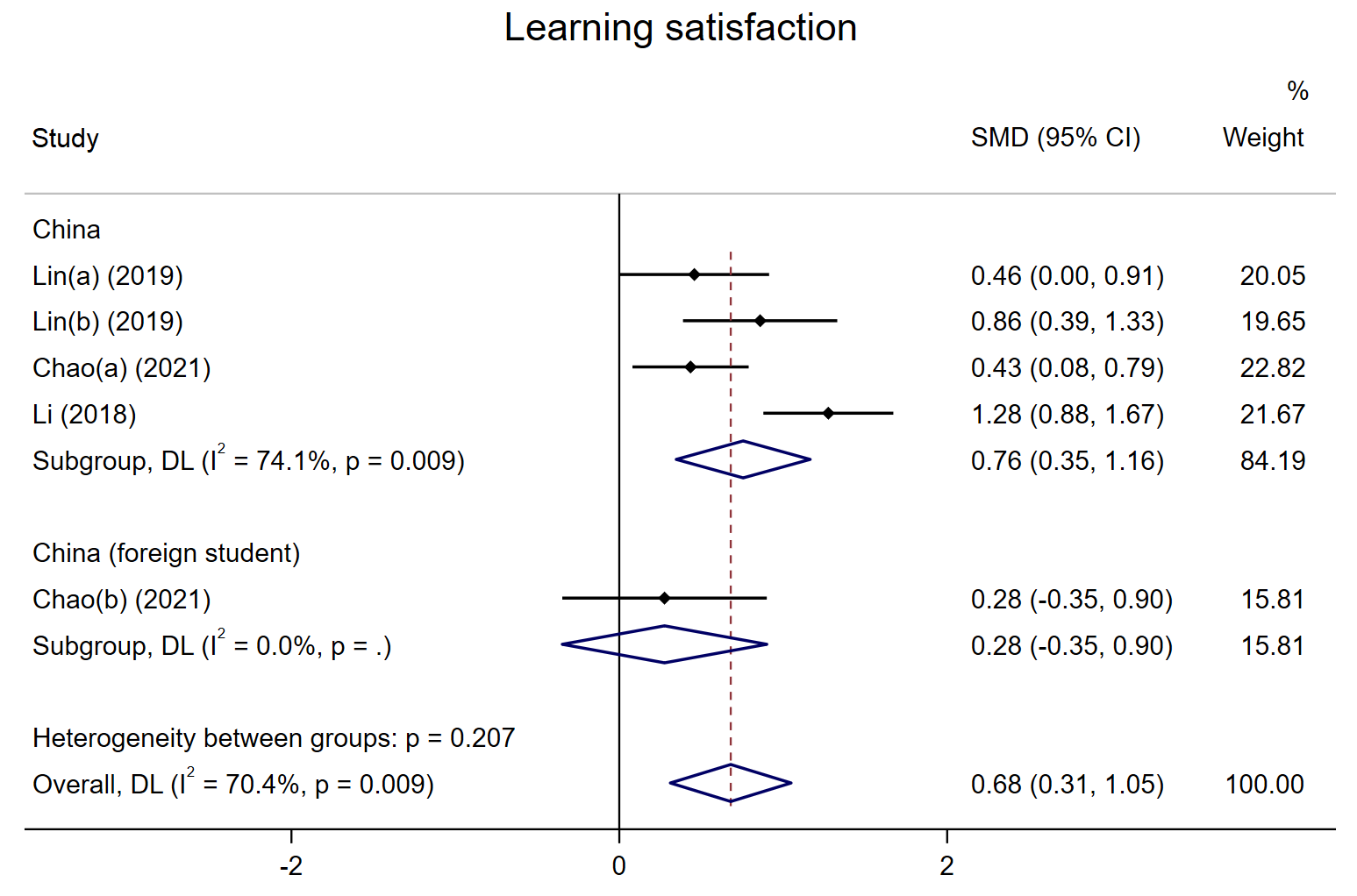


**S9 Fig. The effect of FC versus traditional PE on students' learning satisfaction by country.**


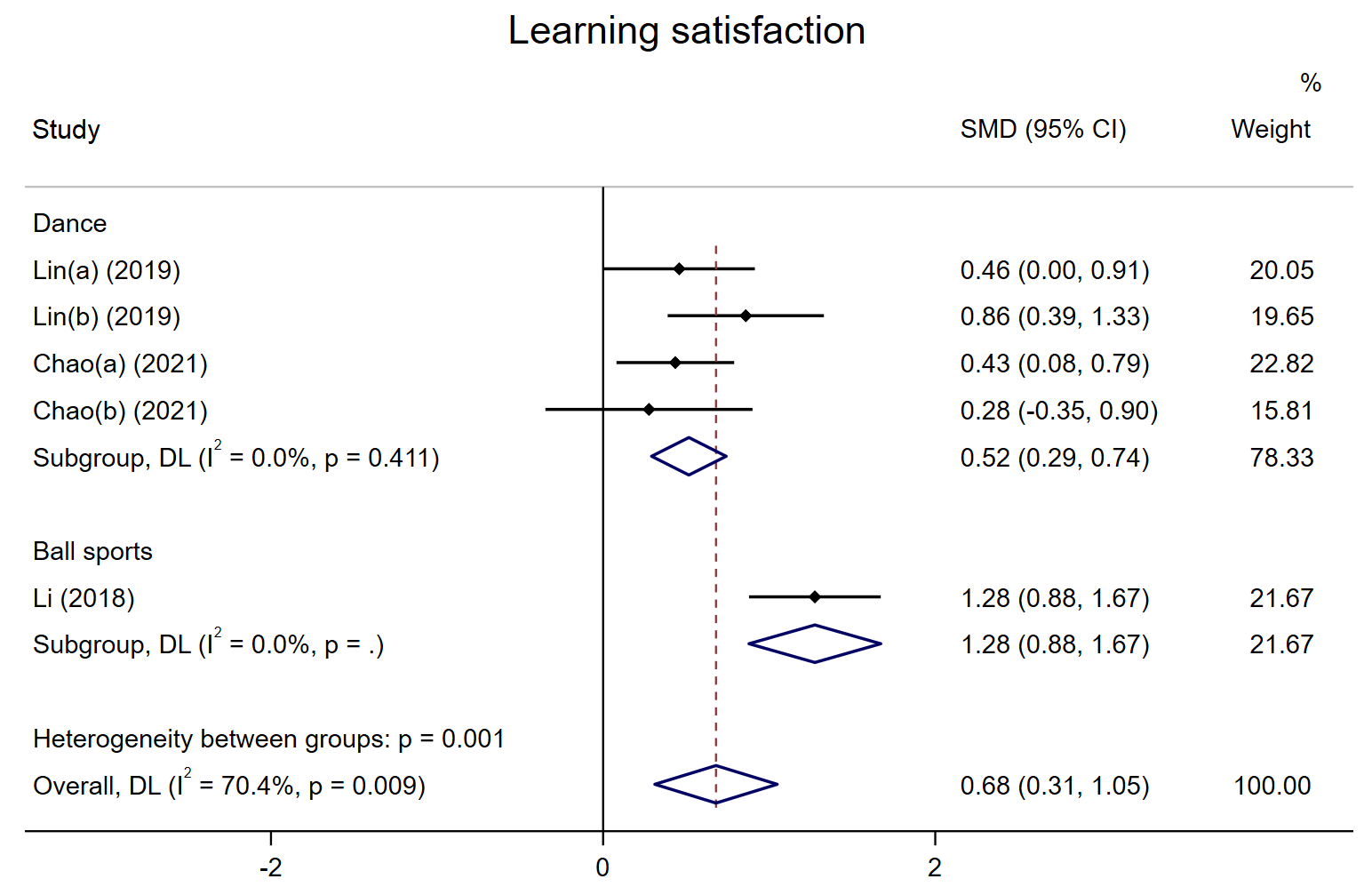


**S10 Fig. The effect of FC versus traditional PE on students' learning satisfaction by PE subject.**


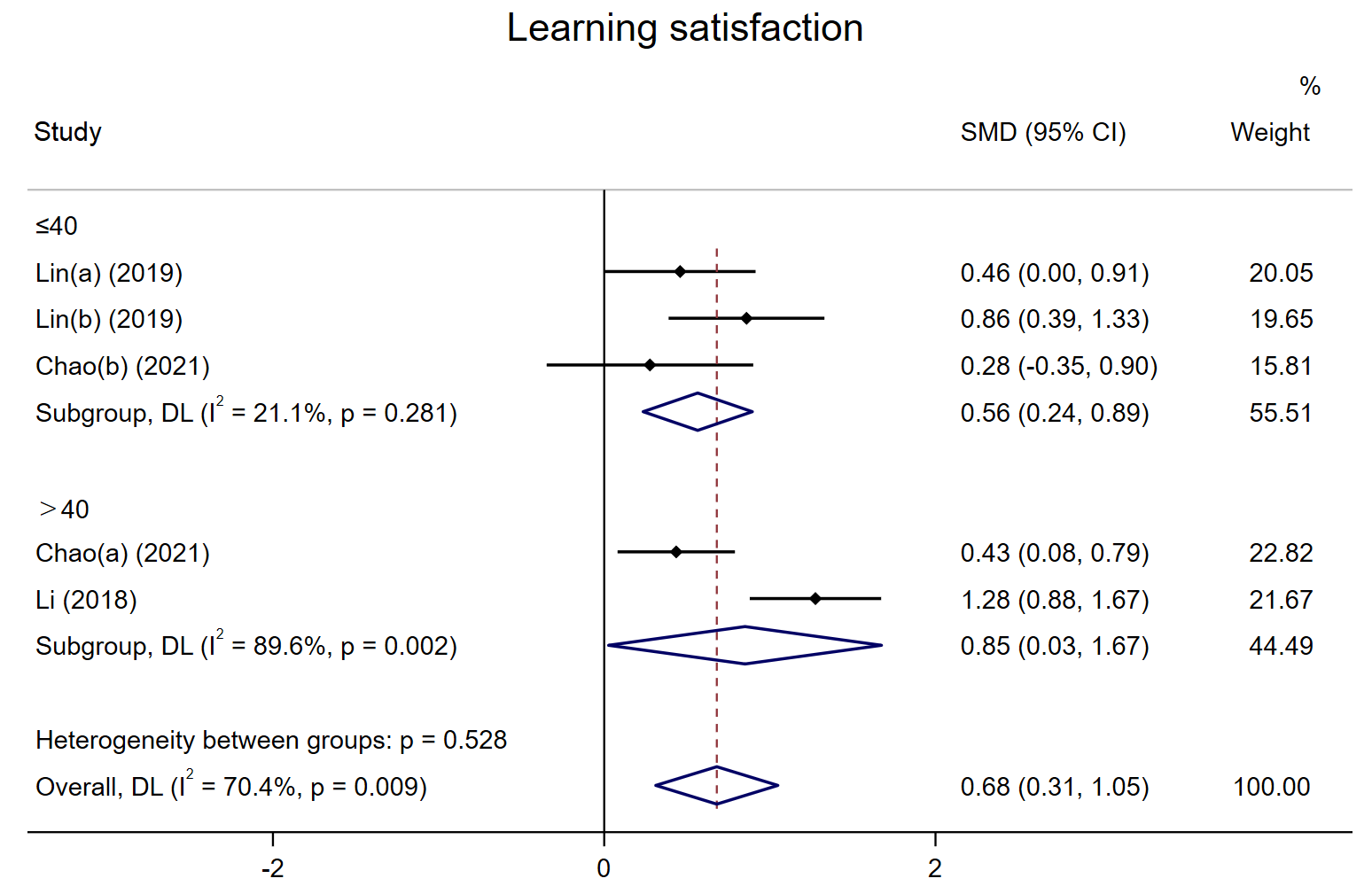


**S11 Fig. The effect of FC versus traditional PE on students' learning satisfaction by class sizes.**





**S12 Fig. Leave-one-out analysis for the association of FC with learning satisfaction.**


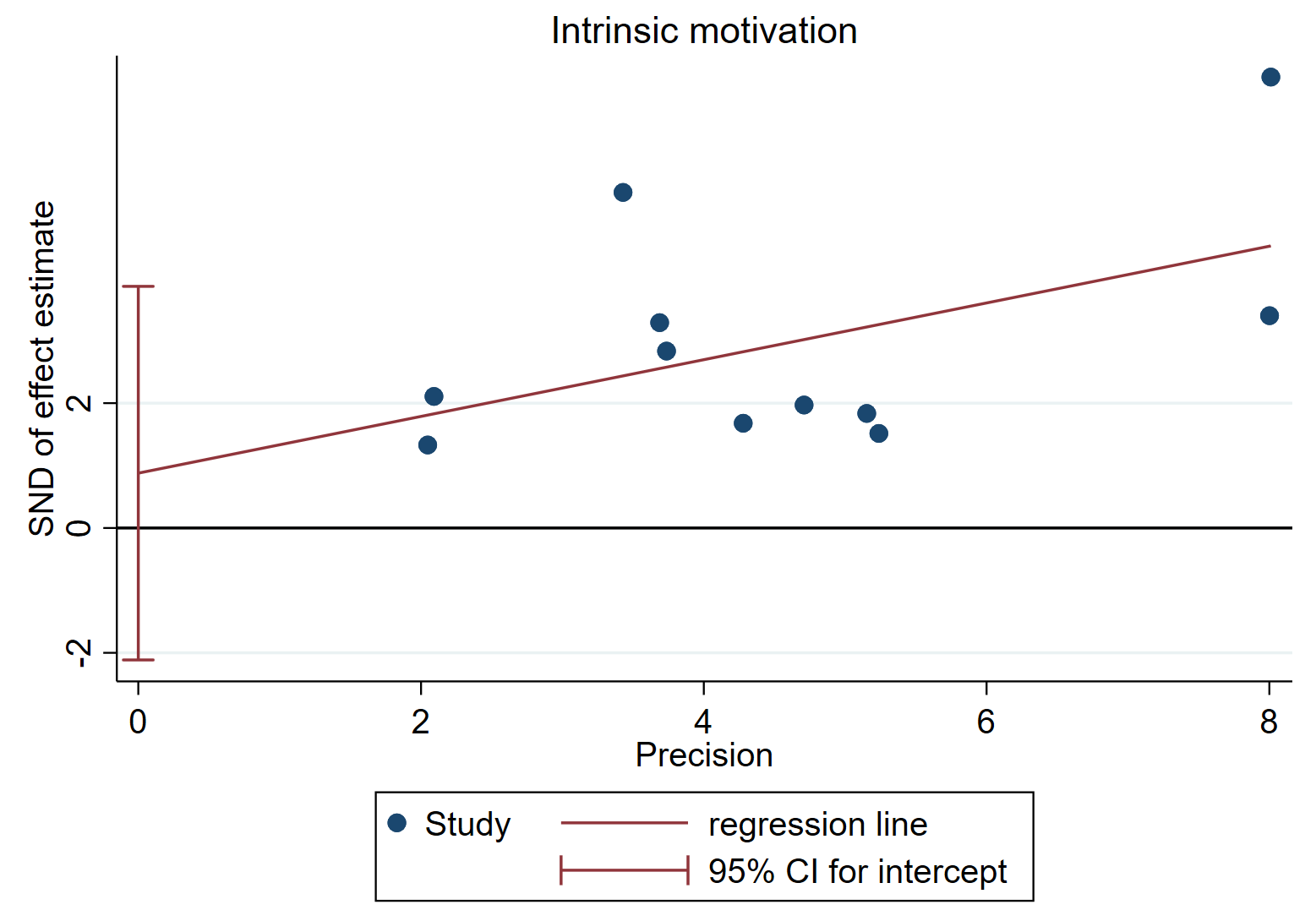


**S13 Fig. Results of publication bias in meta-analyses of intrinsic motivation.**


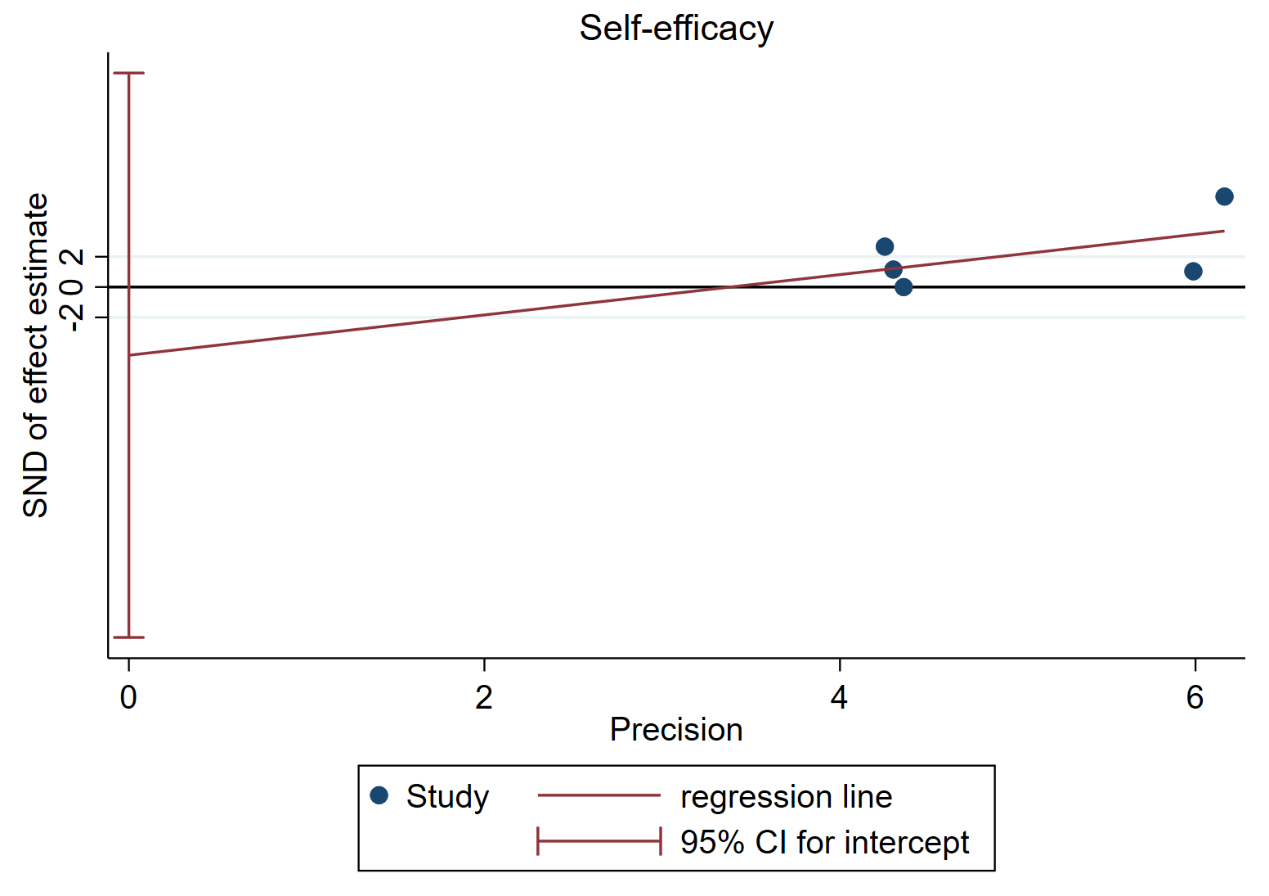


**S14 Fig. Results of publication bias in meta-analyses of self-efficacy.**


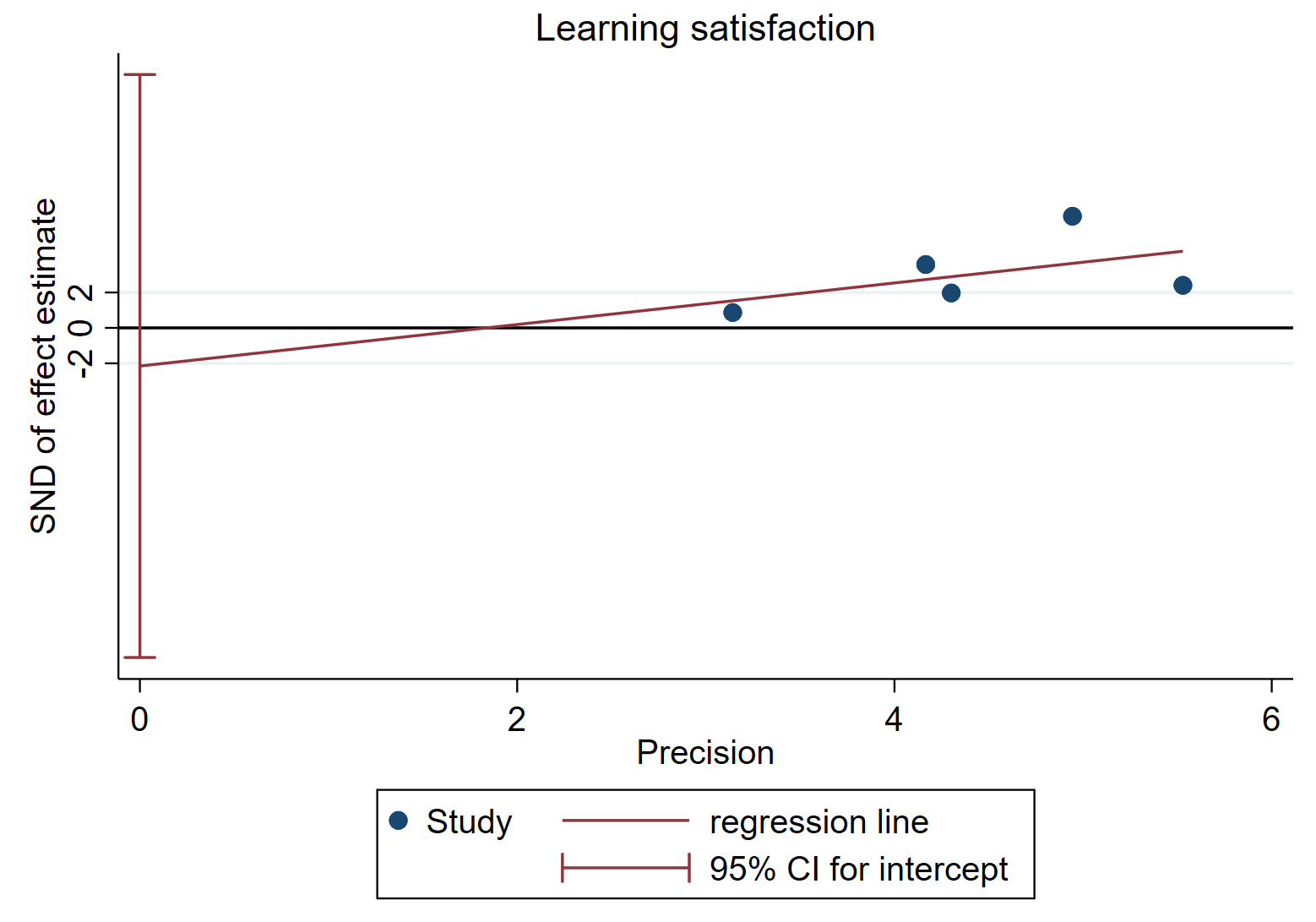


**S15 Fig. Results of publication bias in meta-analyses of learning satisfaction.**
